# An integrative computational approach for obstacle avoidance during action selection

**DOI:** 10.1371/journal.pcbi.1013756

**Published:** 2026-10-26

**Authors:** Shan Zhong, Nader Pouratian, Paul Schrater, Vassilios Christopoulos

**Affiliations:** 1 Alfred E.Mann Department of Biomedical Engineering, University of Southern California, Los Angeles, California, United States of America; 2 Neuroscience graduate program, University of California Riverside, Riverside, California, United States of America; 3 Department of Neurological Surgery, UT Southwestern Medical Center, Dallas, Texas, United States of America; 4 Department of Computer Science and Engineering, University of Minnesota, Minneapolis, Minnesota, United States of America; 5 Department of Bioengineering, University of California Riverside, Riverside, California, United States of America; 6 Department of Neurological Surgery, Keck School of Medicine, University of Southern California, Los Angeles, California, United States of America; 7 Neurorestoration Center, Keck School of Medicine, University of Southern California, Los Angeles, California, United States of America; Indian Institute of Technology Mandi - Kamand Campus: Indian Institute of Technology Mandi, INDIA

## Abstract

Action selection in cluttered environments, where individuals must simultaneously pursue goals and avoid obstacles, presents a significant challenge for the brain. To understand the underlying mechanisms of action selection in such contexts, we propose a computational model that extends stochastic optimal control theory through a novel framework for obstacle avoidance. The model decomposes action selection into a weighted combination of individual control policies, each generated for either target approach or obstacle avoidance. By integrating value information from goals, obstacles, and actions into a unified measure of “relative desirability”, the model dynamically determines the contribution of each policy to the overall action selection process. We evaluated the framework using simulated target-reaching tasks in cluttered environments based on previous human studies. The results showed that the model qualitatively captures key features of human motor behavior, including the influence of obstacle properties on movement trajectories and the transient tendency to initiate movements toward obstacles before avoidance. This work offers new insights into the dynamic interaction between approach and avoidance behaviors, providing a basis for future studies of action selection in more complex and naturalistic settings.

## Author summary

Every day, we make countless movements that require us to reach for something while avoiding obstacles—for example, grabbing a cup without knocking over nearby objects. How the brain selects such actions in cluttered environments remains poorly understood. In this study, we developed a computational model to explore how people plan and control movements when both goals and obstacles are present. In our model, multiple potential actions are prepared at once—some for reaching the goal and others for avoiding obstacles—and then combined based on how desirable each option is at any moment. Using computer simulations of reaching movements, we found that the model qualitatively reproduced key patterns observed in human behavior, including the brief tendency to move toward an obstacle before steering away from it. By showing how continuous competition between approach and avoidance drives can reproduce characteristic behavioral patterns, our work offers a new way to understand how people make rapid and flexible movement decisions in complex, everyday settings.

## Introduction

Consider a hypothetical scenario in which you are invited to a formal dinner and seated at a table with a hot bowl of soup in front of you, a glass of wine just behind it, a salt shaker in the center, and various other items nearby. Each of these objects can act as either a goal or an obstacle, depending on your intentions. For instance, if you want to take a sip of wine, the glass becomes your target. But if someone asks you to pass the salt shaker, the same glass now turns into a potential obstacle. Accidentally knocking it over could create an awkward or even embarrassing situation. The complexity of such interactions increases when certain actions can lead to positive or negative outcomes. Despite the importance of these decisions in everyday life, the mechanisms that allow the brain to select actions that achieve goals while avoiding obstacles remain poorly understood.

Research has shown that goal-directed reaching is not planned in isolation but is continuously shaped by the surrounding visual environment [1,2]. This influence is particularly evident in tasks involving multiple potential targets, where initial reach trajectories are often biased toward a spatial average of the possible goal locations - a phenomenon known as *spatial averaging* [3–5]. Similarly, the presence of non-target objects systematically alters the path of the hand, causing trajectories to deviate even when the objects do not directly obstruct the most efficient route to the goal [6,7].

One theoretical framework that accounts for these dynamic interactions is the “affordance competition hypothesis”, which posits that the brain continuously specifies and prepares multiple potential actions afforded by the environment [8,9]. These partially prepared motor plans then compete for selection in a dynamic process that unfolds over time [10]. This theory is supported by neurophysiological evidence demonstrating the parallel encoding of multiple potential movements in sensorimotor regions [11–14], as well as findings showing that preparing for several possible actions increases motor variability [15]. Within this framework, reaching for a target while avoiding an obstacle can be conceptualized as a competition between a plan to approach the goal and one or more plans to avoid the obstacles.

A key challenge within the affordance competition hypothesis lies in specifying the computational mechanisms that determine how competing motor plans are dynamically weighted and integrated in real-time. This process involves integrating heterogeneous sources of information — such as expected rewards, action costs, and collision risks — which are often expressed in different “currencies” [16,17]. To address this, previous work has proposed converting these disparate signals into a common probabilistic currency that encodes the *relative desirability*, thereby guiding the selection among competing goals [5,17,18]. However, it remains unclear how this framework extends to more complex scenarios involving simultaneous approaching and avoidance behaviors. Early computational models of obstacle avoidance, such as those based on artificial potential fields (APFs) [19,20], treated obstacles as sources of repulsion. Although conceptually elegant in its simplicity, the classical APF approach suffers from notable limitations, most prominently the tendency to get trapped in local minima [21,22]. In robotics, these challenges have motivated both the refinements to the potential field formulation [23,24] and the emergence of more sophisticated techniques broadly classified as heuristic, predictive, or learning-based approaches [25]. Reinforcement Learning (RL), for instance, enables agents to learn optimal obstacle avoidance policies through trial-and-error iterations with their environment, offering enhanced adaptability to complex and dynamic contexts [26,27]. Other modern techniques, including predictive methods such as model predictive control (MPC) and various swarm-based optimization algorithms, further expand the ability to solve obstacle avoidance problems that traditional methods struggle to manage [25]. While these advanced methods provide powerful solutions for autonomous navigation, their primary objective is often optimal performance in robotic systems, not necessarily biological plausibility. The goal of modeling human motor control is different; it seeks to understand the specific computational principles that give rise to observed human behaviors. For instance, the tendency for reaching trajectories to sometimes initially deviate towards a non-target object before curving away suggests a more complex integration of competing motor plans than simple repulsion or pure optimization would predict [2].

In the current study, we take a step towards a unified computational theory for action selection by extending the probabilistic framework introduced by Christopoulos et al. [17] - originally developed for multi-target choice - to the specific problem of reaching in cluttered environments. In our model, the final motor command is formulated as a dynamically weighted mixture of policies for target approach and obstacle avoidance, with each policy generated by a stochastic optimal controller [28]. We show this model qualitatively captures key features of human reaching behavior, including the characteristic initial deviation of trajectories toward an obstacle. More broadly, this framework provides a principled means to describe the continuous competition between approach and avoidance drives, opening new avenues to investigate the neural computations that govern goal-directed movements in complex and cluttered environments.

## Results

### A computational model for selecting action plans in complex environments

The architecture of the model is a series of optimal control schemes associated with different targets/obstacles, as is shown in Fig 1. In stochastic optimal control theory, the objective is to determine a control policy that minimizes a cost function representing the trade-off between effort and goal attainment. Each optimal control scheme generates a specific action plan (policy) $\pi_{j}(x_{t})=u_{j}=[u_{1},u_{2},...u_{T_{j}}]$, which defines a sequence of motor action plans *u* starting from the current state $x_{t}$ and directed towards the desired goal *j*. The performance of a policy is evaluated by a cost function $V_{\pi_{j}}(x_{t})$, which computes the expected cumulative cost (i.e., effort) required to reach goal *j* from the current state while minimizing deviation from the desired final state.

**Fig 1:**
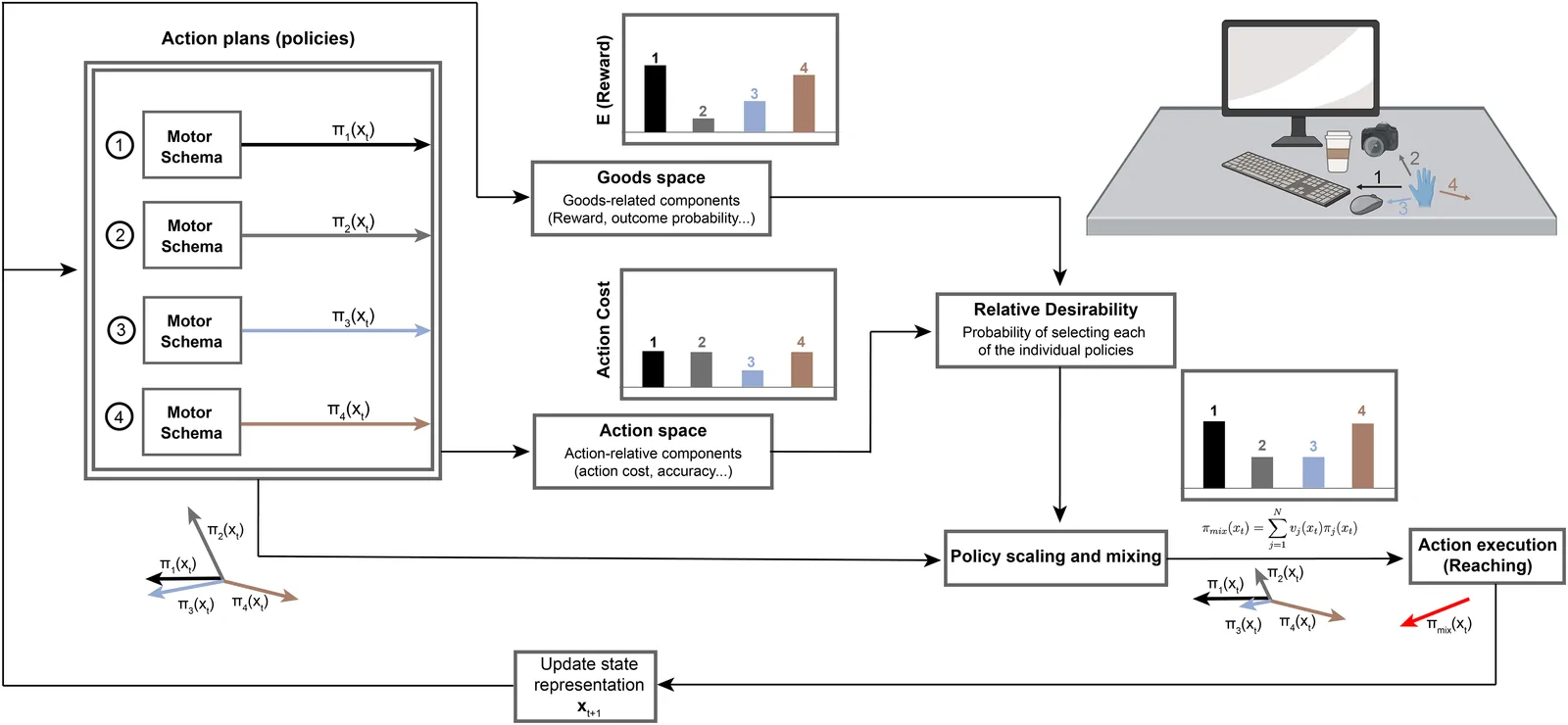
Model architecture. The model consists of a series of optimal control schemes associated with different targets/obstacles. In this hypothetical scenario, a hand is positioned over a desk with different objects, among which three of them are potential targets (a keyboard, a computer mouse, and a camera), and one is a stationary obstacle (a coffee cup). These objects are located at different distances from the hand’s current state $x_{t}$. Under these conditions, the motor schemas associated with each of these four objects are activated and generate four action plans $\pi_{1}$ to $\pi_{4}$. Note that $\pi_{4}$ is negated since it corresponds to the obstacle. At each time t, the desirabilities of each of these action plans are computed based on their action-related components and goods-related components. Then these two values are combined to make an overall relative desirability. The final policy that the hand executes is a weighted mixture of the individual policies weighted by the relative desirability values. Created in BioRender. Zhong, **S.** (2026) https://BioRender.com/ua6pm7l.

Consider the reaching scenario in Fig 1, where a hand (at state $x_{t}$) is positioned over a desk containing different target objects (a keyboard, a mouse, and a camera) located at distinct positions and distances, along with a stationary obstacle (a coffee cup) that must be avoided during reaching movements. Under these conditions, the optimal control schemes associated with the targets and obstacle are simultaneously activated, generating four action plans ($\pi_{1}$ to $\pi_{4}$) to move towards the targets and obstacle. The action plan directed toward the obstacle is inverted (negated) to construct a policy for avoiding it. Each action plan is associated with an action-related component, reflecting the effort required to execute that plan. For instance, it is typically less “costly” for the hand to move directly towards a target than to follow a curved trajectory. The targets are also associated with goods-related components, such as expected utility and probability of successful acquisition. For instance, reaching for the keyboard (the primary task object) offers higher utility than reaching for the mouse (a secondary object) in the current scenario. Furthermore, the obstacle is associated with obstacle-related components, such as the potential cost incurred upon collision (e.g., spilling coffee) and the probability of such a collision occurring based on the hand’s trajectory and the cup’s proximity. Consequently, the hand exerts greater effort to maintain safe clearance from the coffee cup when its position creates a higher collision risk.

In this study, we consider a simplified special case of the general goods-related formulation. Because the simulations involve only a single target, reward comparisons across competing targets reduce to a constant. In addition, obstacle-related influences are represented through the probability of collision, such that the goods-related term effectively reduces to the probability of avoiding a collision. During the reach, however, the effort cost, the probabilities of reaching the target, and the probability of avoiding an obstacle all change dynamically. These evolving quantities must therefore be integrated online into a common currency, termed *relative desirability* [17]. The relative desirability integrates information from both the action and goods space, quantifying the momentary preference for each candidate action plan. The resulting policy is then formed as a weighted mixture of action plans, where the contribution of each plan is determined by its relative desirability.

### Collision avoidance reshapes relative desirability distributions

To better understand how action cost and collision probability influence reaching behavior during obstacle avoidance, we visualized the spatial distribution of relative desirability under two conditions: (1) an action cost-driven scenario, in which only the action-related component is considered; and (2) a comprehensive scenario, which incorporates both the action-related and the goods-related (collision avoidance) components. For computational tractability and clarity of visualization, we focused the simulations on scenarios with a single target and a single obstacle, though the framework could generalize to multiple targets and obstacles as described in the Methods section. In the action cost-driven scenario, following the approach described in [17], we projected the four-dimensional desirability map (position-velocity space) onto two dimensions by constraining all the trajectories to originate from the position (0,0) with zero initial velocity. We defined a circular sampling region centered at (30,30) - i.e., the target location, with a radius of 75% of the origin-to-target distance, and allowed the trajectories to arrive at 359 evenly distributed spatial positions along the circle. At each position, the velocity was set radially outward from the origin through that spatial position, and speed was matched to that of an unobstructed optimal reaching movement at 75% of completion. This representative value was chosen for visualization purposes. The obstacle was positioned at (15,15). From each of these 359 positions on the circle, we generated simulated reaching trajectories toward the target while accounting for the obstacle. To incorporate variability, ten trials were simulated for each starting point. Representative trajectories are illustrated as arrows in Fig 2. Next, we discretized the space into a grid and computed multiple action-cost measures for each state, including the cost-to-go for reaching the target and avoiding the obstacle, as well as the relative desirability values associated with both. These computations produced a comprehensive state-space map that captures target-directed reaching behavior and obstacle avoidance (Fig 2). The spatial distributions of cost and relative desirability were then visualized using heat maps and vector fields (Fig 2).

**Fig 2:**
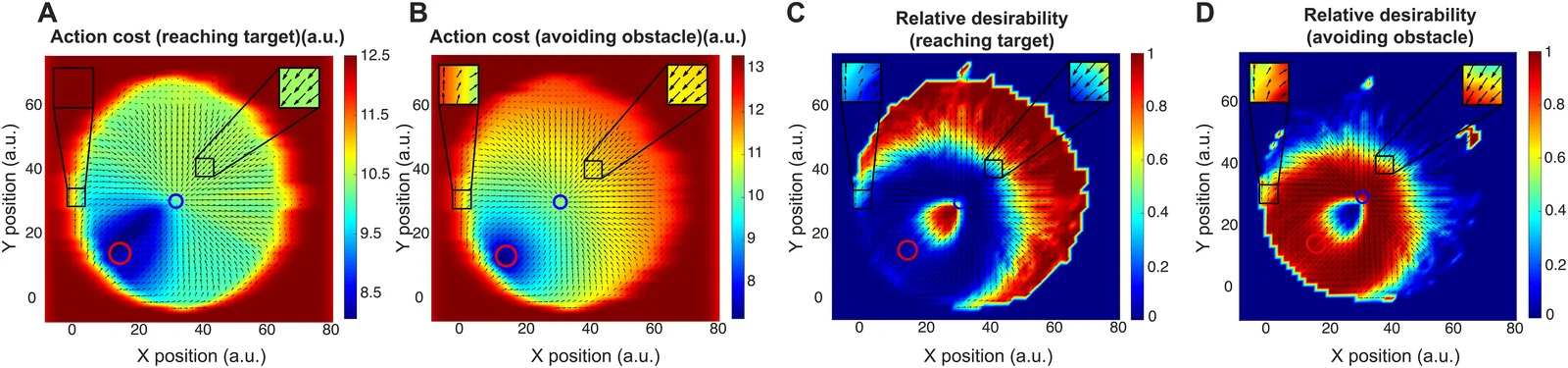
Action cost-driven relative desirability in obstacle avoidance. **(A)** Heat map of the action cost for reaching the target (blue circle) from different states. Warmer colors (reddish hues) represent higher-cost states, while cooler colors (blueish hues) represent lower-cost states. Insets: close-up view of representative positions on the map, where the black arrows show the average velocity at the given state. **(B)** Same as **(A)**, but for avoiding the obstacle (red circle). **(C)** Heat map of the relative desirability for reaching the target. Warmer colors represent higher desirability, while cooler colors represent lower desirability. **(D)** Same as **(C)**, but for avoiding the obstacle.

As shown in Fig 2A, the action cost for reaching the target is lowest in the bottom-left region, where minimal deviation in movement direction is required. In contrast, Fig 2B illustrates that the action cost associated with avoiding the obstacle is lowest in the vicinity of the obstacle. This counterintuitive outcome arises because the obstacle-avoidance policy is defined as the negation of the policy for reaching the obstacle, effectively creating an “anti-target” at the obstacle’s location. When the two action-cost maps are integrated to compute the relative desirability (Fig 2C and 2D), the resulting spatial distribution of policy weights appears more realistic. The desirability for reaching the target is high when the current state is distant from the obstacle (Fig 2C), consistent with intuitive expectations. However, the model still produces an unrealistic “rim” of high obstacle-avoidance desirability surrounding the target (Fig 2D). This occurs even when the trajectory is directed away from the obstacle and is close to the target, conditions under which avoidance behavior would be unlikely in natural settings. When trajectory simulations are generated under this cost-only formulation, the elevated avoidance desirability near the target can induce early deviations away from the target or, alternatively, failure to account for the obstacle, resulting in collisions and qualitatively implausible behavior (see Supplementary Material, S1 Fig). This discrepancy shows a limitation of relying solely on action cost, as it fails to account for the spatial relationship between the current movement direction and the position of the obstacle.

In the comprehensive scenario, we went beyond computing the cost of reaching the target and avoiding the obstacle, by also estimating the collision probability using an expanded obstacle representation. We introduced an expanded *collision zone* surrounding the actual obstacle, defined by two diameters: an inner diameter corresponding to the physical size (i.e., diameter) of the obstacle, and an outer diameter specifying the tolerance boundary within which a potential collision is still considered likely. Collision probability is computed based on the position of the hand relative to these boundaries. Points within the inner diameter are assigned 100% collision probability. For points between the inner and outer diameters, the probability decreases linearly from 100% to 0%, a simplification chosen for analytical transparency whose impact on qualitative predictions is evaluated below. Fig 3 applies the same visualization approach used in the action cost-driven scenario, but now incorporates the collision probability, resulting in a more comprehensive description of the obstacle avoidance task. In Fig 3A, we illustrate the collision probability, which reveals two distinct *collision cones*: one centered around the obstacle, as expected, and a second surrounding the target. The latter captures trajectories that, while aimed at the target, approach the obstacle dangerously closely. Within each cone, the collision probability peaks at the center and gradually decreases towards the periphery. By integrating these collision cones with the previously computed action costs presented in Fig 2, we observe remarkable changes in the relative desirability landscape (Fig 3B and 3C). Specifically, trajectories that approach or pass in close proximity to the obstacle are assigned lower desirability. The resulting maps illustrate how collision probability reshapes relative desirability within the model.

**Fig 3:**
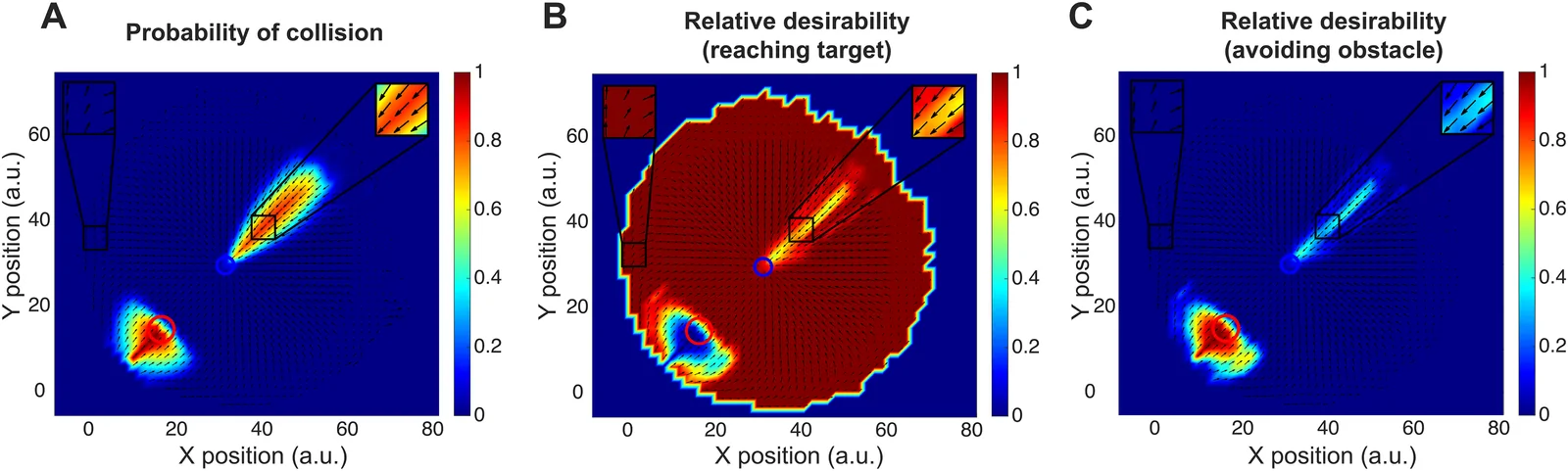
Comprehensive relative desirability in obstacle avoidance. **(A)** Heat map showing the probability of collision, where warmer colors denote higher probabilities and cooler colors represent lower ones. Insets: close-up view of representative positions on the map, where the black arrows show the average velocity at the given state. **(B)** Heat map of the relative desirability of reaching the target, taking into account both action costs and collision probability. Warmer colors represent higher desirability. **(C)** Same as **(B)**, but illustrating the relative desirability associated with avoiding the obstacle.

### Relative desirability reproduces qualitative features of reaching behavior during obstacle avoidance

To evaluate whether the model qualitatively reproduces characteristic features of human reaching behavior, we simulated reaching trajectories across multiple obstacle configurations using the closed-loop procedure summarized in Algorithm 1.

**Algorithm 1 Simulated Trajectory Generation (Conceptual Overview)**

**Input:** Starting position, target location, obstacle location, risk aversion factor

**Output:** Simulated reaching trajectory

1: Compute optimal control policy for target approach

2: Compute optimal control policy directed toward obstacle

3: **while** target not reached **do**

4:  Generate motor commands: one toward target, one away from obstacle

5:  Evaluate effort cost of each command at current state

6:  Compute relative desirability using effort costs and current collision probability

7:  Mix commands weighted by desirability and risk aversion factor

8:  Execute mixed command; apply signal-dependent motor noise

9:  Update collision probability from executed trajectory

10:  Advance to next state

11: **end while**

We focus on two key scenarios: one in which the obstacle lies directly in the path to the target, and another where the obstacle is nearby but does not obstruct the direct path. Fig 4 illustrates the first scenario, where an obstacle is positioned between the starting point and the target. To assess how the obstacle size influences behavior, we systematically increase the diameter of the obstacle across trials.

**Fig 4:**
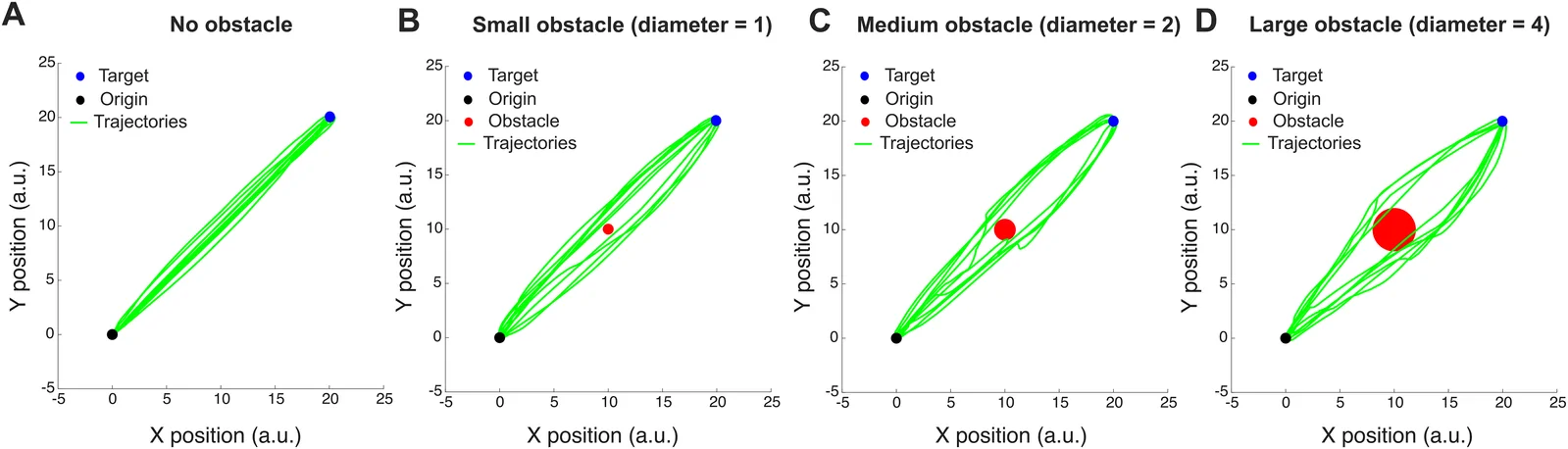
Simulated reaching trajectories with varying obstacle sizes along the direct path. Simulated reaching trajectories (green lines) for four conditions: **(A)** No obstacle, (B) small obstacle (diameter = 1), (C) medium obstacle (diameter = 2), and (D) large obstacle (diameter = 4). The origin is indicated by a black dot, the target by a blue dot, and obstacles by red circles.

As shown in Fig 4, the model produces distinct reaching behaviors across varying conditions. In the absence of an obstacle, trajectories to the target are direct and efficient. As an obstacle is introduced and its size progressively increases, the reaching movements begin to deviate, curving away from the obstacle’s location. For sufficiently large obstacles, the model occasionally predicts collisions. These predictions are qualitatively consistent with natural human behavior, where larger obstacles tend to elicit wider avoidance paths and, in some cases, result in contact.

To assess the effect of the collision-probability decay function, we repeated the simulations in Fig 4B–4D using exponential and Gaussian CDF-based decay profiles (S2 Fig). The trajectories were qualitatively similar across the three tested functions, which share the same boundary conditions and differ only in their intermediate profiles.

For all subsequent simulations, we retained the linear decay function for simplicity.

Fig 5 illustrates the second scenario, in which the obstacle is positioned near - but not directly obstructing - the reaching path towards the target. We simulated 100 trials for each condition, with the obstacle placed either to the left (Fig 5A) or to the right (Fig 5B) of the direct path from the origin to the target. Interestingly, the simulations often produced trajectories that initially deviated towards the obstacle - an effect consistent with previous observations in human studies [1,29]. In some cases, these trajectories subsequently curved around the obstacle before continuing towards the target. This pattern arises from the interplay between signal-dependent motor noise and the temporal structure of collision feedback. At movement onset, collision probability is low and the policy mixture is dominated by the target-approach policy, therefore, noise-driven displacement toward the obstacle goes initially uncorrected [28]. As the trajectory enters the collision zone, collision probability rises and avoidance gains weight, producing the subsequent curving behavior. The effect is therefore trial-variable, consistent with evidence that toward-obstacle deviations in human reaching depend on the state of motor competition at movement initiation [2]. For visualization clarity, we separated these trajectories from the rest and computed their standard errors independently. In other instances, the trajectories deviated away from the obstacle even when it did not directly block the path, resulting in a rightward bias when the obstacle was on the left side (Fig 5A) and a leftward bias when it was on the right side (Fig 5B), consistent with findings from prior human studies [7,30]. These patterns are consistent with the subtle influence of nearby obstacles on human reaching behavior, in which individuals tend to avoid the obstacle even when it does not directly interfere with the most efficient path to the target.

**Fig 5:**
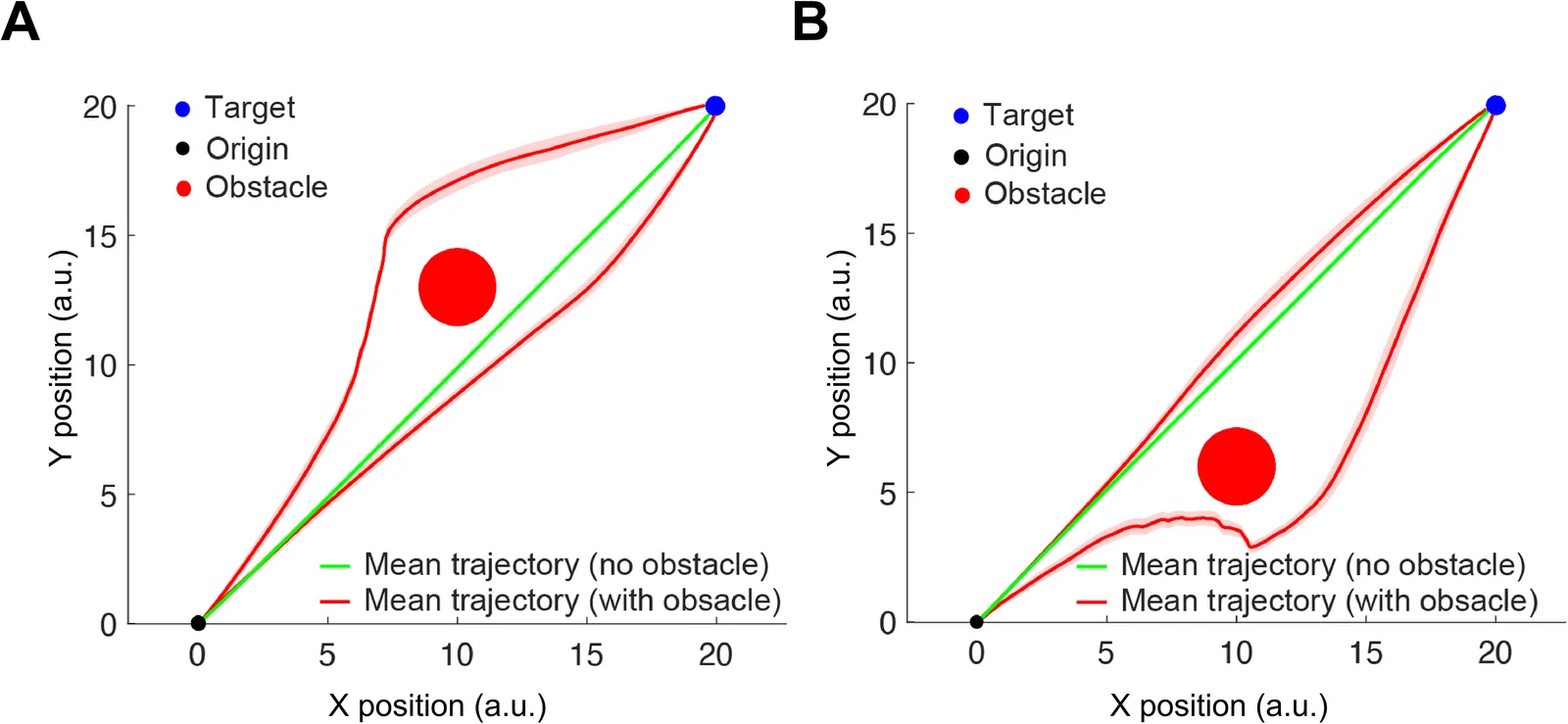
Simulated reaching trajectories with nearby obstacles. Mean simulated reaching trajectories with (red lines) and without (green lines) an obstacle (red circle) when reaching toward a target (blue circle). Shades represent standard error of the simulated trajectories. **(A)** Obstacle located to the left of the direct path. **(B)** Obstacle located to the right of the direct path.

Furthermore, we examined the performance of our model in more dynamic environments where the target location changes mid-flight. Fig 6 illustrates two such scenarios. In Fig 6A, the target jumps to a new position during the reaching movement, and we simulated two conditions: one with an obstacle (red circle) positioned along the direct path to the new target location, and one without any obstacle (green trajectories). In Fig 6B, we examined a similar target-jump scenario under three conditions: a no-obstacle condition (green trajectories), and two separate obstacle configurations (obstacle 1 - red circle and obstacle 2 - blue circle), in which an obstacle was positioned at different nearby locations that did not directly block the path to the new target location. Across all conditions, the model successfully generated trajectories that redirected toward the new target location. Importantly, trajectories deviated away from the obstacle, whether the obstacle directly obstructed the path (Fig 6A) or was merely nearby (Fig 6B). These simulations indicate that the model qualitatively reproduces the flexible, adaptive characteristics of human motor control in dynamic environments, preserving obstacle-avoidance behavior when goals shift during movement execution.

**Fig 6:**
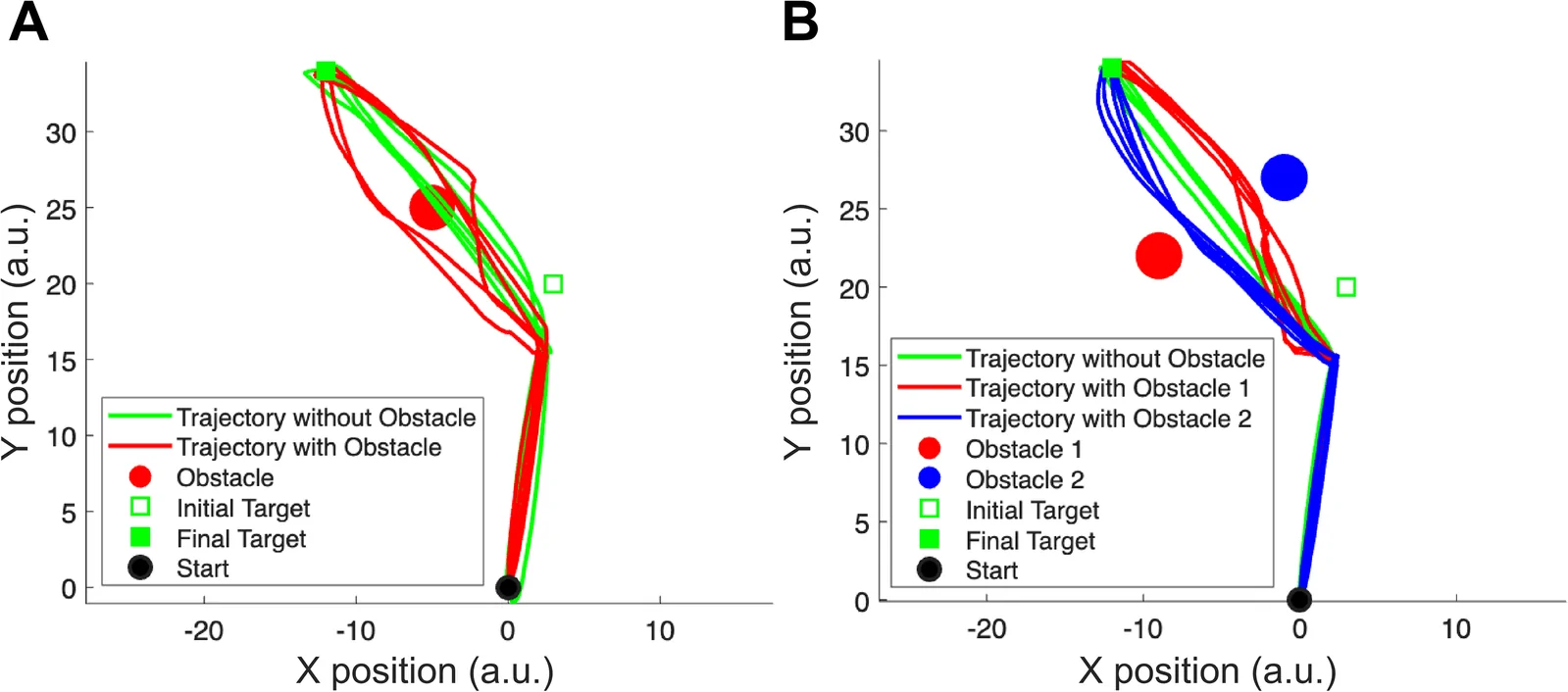
Simulated reaching trajectories in dynamic environments with mid-flight target changes. **(A)** Reaching behavior when the target jumps to a new location during movement, with (red) and without (green) an obstacle blocking the direct path to the final target. **(B)** Similar to **(A)**, but with three conditions: no obstacle, and two different obstacle configurations (obstacle 1 or obstacle 2) positioned nearby but not directly blocking the path to the final target.

## Discussion

### General

The ability to navigate in cluttered environments while simultaneously approaching targets and avoiding obstacles is fundamental to everyday behavior, yet the underlying computational mechanisms remain poorly understood. Traditional computational approaches, such as APFs, treat obstacles as sources of simple repulsion but suffer from limitations like local minima trapping [21,22]. While modern robotics has developed sophisticated solutions [25,27], these approaches prioritize optimal performance over biological plausibility. The current study addresses this challenge by extending the probabilistic framework of Christopoulos et al. [17] from multi-target choice scenarios to the specific problem of reaching in cluttered environments. Building upon the affordance competition hypothesis [8,9] and previous work on action-selection and value integration [17], we developed a model that integrates action-related costs and collision probability, with target reward held constant in the present simulations. The model decomposes the complex problem into a weighted mixture of individual control policies, each generated by a stochastic optimal control system to either move towards a target or avoid an obstacle. The relative desirability of each policy serves as a common currency for comparing and integrating the value information from disparate sources [17], consistent with the notion of a common neural currency for decision-making [31].

The results demonstrate that the model qualitatively captures key aspects of human reaching behavior in the presence of both targets and obstacles, such as the tendency to deviate from the direct path to the target, as well as the occasional deviation towards the obstacle before curving around and turning to the target. Furthermore, the framework predicts a decrease in the desirability of movements toward a target when an obstacle is located immediately beyond it along the same movement direction—that is, when the obstacle does not impede the reach itself but is positioned just past the target. These results align with previous findings in human studies [3,32,33] and highlight the model’s ability to account for the spatial relationship between the current movement state and the obstacle. Within the model, these trajectory patterns can arise from the dynamic integration of competing motor plans. In particular, toward-obstacle deviations occur because the avoidance policy carries little weight at movement onset, paralleling the interpretation of Welsh and Elliott [2] that such deviations reflect incomplete inhibition of competing responses at the time of initiation. This logic is also consistent with the minimal intervention principle [28], which states that early displacement toward the obstacle is task-irrelevant and therefore goes uncorrected until the hand enters the collision zone, a pattern supported by evidence that hand-path variability does not decrease near obstacles [34].

### Model characteristics and biological plausibility

It is important to acknowledge that our model does not provide a computationally optimal solution to obstacle avoidance. The “policy negation” approach — i.e., computing the policy to reach an obstacle and then inverting it — serves as an approximation rather than the result of directly optimizing an avoidance cost function. This negation step is necessary because stochastic optimal control inherently requires a well-defined end goal, whereas obstacle avoidance lacks a specific endpoint to move toward. Our aim is to construct a biologically motivated computational model that qualitatively reproduces characteristic features of human reaching behavior, which is inherently variable and often departs from optimality [35]. Such deviations likely reflect fundamental constraints of neural computation, including uncertainty, processing delays, and risk sensitivity. Accordingly, the value of the model lies not in generating optimal trajectories, but in reproducing realistic behavioral phenomena, including seemingly inefficient patterns such as the initial deviation of the hand toward an obstacle before subsequently curving away from it [2].

Policy negation is motivated by a conceptual analogy with neural transformations of movement direction. Transformations that reverse the direction of a planned movement are known to occur in the nervous system, as demonstrated in anti-saccade [44] and anti-reach [45,46] tasks, the latter arising through gain modulation of directional tuning in parietal and premotor cortex. Similarly, obstacle avoidance engages premotor processes that are at least partly dissociable from those supporting direct reaching. Neurons in dorsal premotor cortex encode both target direction and the initial movement direction required to circumvent an obstacle [47], while premotor lesions selectively impair obstacle avoidance while largely sparing direct reaches [48]. These observations provide biological motivation for distinguishing target-approach and obstacle-avoidance processes in the model.

At the same time, we do not interpret these findings as evidence that the brain literally implements obstacle avoidance by inverting a control policy. The anti-saccade and anti-reach studies involve discrete movements directed toward a defined endpoint, whereas our model applies negation to a continuous control policy in the absence of a terminal state. Thus, policy negation should be viewed as a computational abstraction inspired by known neural transformations of movement representations, rather than as a direct account of the underlying neural mechanism. A more biologically grounded implementation may instead emerge from competitive interactions among candidate actions within a shared neural substrate, as proposed by dynamic neural field (DNF) and affordance competition models of action selection [8,49].

Overall, our model provides a computational framework for investigating how obstacle avoidance may emerge from the dynamic competition and integration of multiple motor plans, rather than through the optimization of a single global cost function. Although this approach differs conceptually from traditional obstacle-avoidance algorithms, it is not intended to diminish the value of methods such as artificial potential fields (APF), reinforcement learning (RL), or model predictive control (MPC), which have proven highly effective for their intended applications in robotics, autonomous navigation, and engineering control systems [23,25–27]. These algorithms are designed to maximize performance according to predefined objectives, whereas our model is intended to provide a computational account of qualitative patterns in human motor behavior and generate experimentally testable hypotheses regarding the neural computations that may underlie obstacle avoidance.

### Limitations and future perspectives

By qualitatively capturing key aspects of human obstacle avoidance behavior, the model also opens new avenues for refining and extending its underlying mechanisms. One such aspect is the treatment of collision probability, which is currently computed using collision cones constructed from concentric circular boundaries surrounding each obstacle. This representation involves two simplifications. First, the tolerance boundary is selected to reflect the spatial scale of the simulated task and the physical size of the obstacle, rather than systematically derived from data. Experimental work has shown that safety margins during reaching vary systematically with obstacle geometry, size, and material properties [7,33,36], and that such variation can be formalized through perceived-risk models [37–39]. Second, the circular boundary assumption does not capture the influence of obstacle shape on avoidance behavior, which has been shown to be significant [36,40]. However, both simplifications reside entirely within the collision probability computation. The relative desirability framework that integrates collision probability with action costs is structurally independent of how the boundary is specified or shaped. This modularity means that obstacle-dependent boundaries, for example, derived from perceived-risk formalisms [37], or collision cones generalized to objects of arbitrary shape [41], could replace the current implementation without requiring changes to the rest of the model architecture. Importantly, the current implementation represents a simplified case in which the reward-related term reduces to a constant because the simulations involve a single target, while obstacle-related costs are captured through collision probability. The broader framework, however, maintains distinct reward and loss terms, enabling the incorporation of differential target rewards and obstacle-specific loss magnitudes as a natural extension of the current formulation.

Moreover, the comparison in S2 Fig addresses the tested collision-probability decay functions, but not general parameter sensitivity. A comprehensive sensitivity analysis of the risk aversion parameter α, tolerance boundary, controller parameters, and other model parameters has not been performed. In particular, α was treated as a fixed value in our simulations. However, evidence suggests substantial individual differences in risk-taking behavior [42,43], showing that this parameter likely varies across subjects and contexts. Investigating how α relates to behavioral or neural markers could provide insights into the mechanisms underlying individual differences in risk-taking.

Furthermore, while our systems-level model reproduces characteristic behavioral patterns in environments with both static and dynamically changing obstacles, extending it to more complex contexts would offer a more rigorous assessment of its generalizability. Although the mathematical formulation accommodates multiple targets and obstacles (Eq 4), the present simulations were intentionally restricted to simpler configurations in order to isolate and characterize the fundamental approach-avoidance computations introduced by the framework. In addition to static conditions, we also considered a dynamic scenario in which the target changed location and a new obstacle appeared during movement. Behavioral performance in more complex cluttered environments containing multiple targets and obstacles remains to be demonstrated.

The present qualitative findings also do not establish whether our model provides a better quantitative account of human obstacle-avoidance behavior than alternative models. Systematic quantitative comparisons with human reaching behavior, calibrated to the specific conditions of individual experiments (e.g., physical setup, movement kinematics, and obstacle geometry), will be essential for evaluating predictive accuracy, refining model parameterization, and systematically assessing the sensitivity of model predictions to assumptions such as the collision-zone boundary and risk-aversion weighting. Quantitative metrics such as collision rate, maximum lateral deviation from the straight-line path, initial deviation angle toward the obstacle, minimum clearance distance between the hand trajectory and the obstacle, and trajectory curvature could serve as concrete benchmarks for such comparisons. Such behavioral validation will also pave the way for tackling more complex real-world conditions involving multiple moving obstacles and goals, continuous goal adjustments, and predictive planning, which require a framework capable of integrating desirability principles within a dynamic architecture. Recent work has shown that the relative desirability framework, when combined with DNF theory, could successfully and effectively model both the underlying behavioral and neural mechanisms of action selection, stopping and switching of actions [18,49], suggesting that our principles of desirability and dynamic policy integration have the potential to naturally extend to cluttered, dynamic environments. Nevertheless, the biological interpretation of the present model remains a hypothesis to be tested through neurophysiological recordings and perturbation experiments. Electrophysiological recordings in premotor, motor, and parietal cortices, as well as in subcortical regions such as the subthalamic nucleus (STN), which is involved in action reprogramming and movement pausing, could provide valuable insights into the predictions of the proposed model.

## Conclusion

In conclusion, the current study presents a biologically motivated computational model that extends probabilistic action selection principles to obstacle avoidance scenarios. By dynamically updating the relative desirability of individual control policies, the model provides a computational hypothesis for how actions may be selected in the presence of both goals and potential obstacles. Future studies on the neural mechanisms of approach–avoidance behavior, informed by our computational framework, will be vital for elucidating how the brain achieves flexible and adaptive control in complex settings.

## Methods

### Model architecture

The model is comprised of a series of stochastic optimal control systems, each of them attached to an individual goal or an obstacle (Fig 1). Each controller *j* generates an optimal policy $\pi_{j}$, which is a mapping between a current state and optimal actions, and an action cost function $V_{\pi_{j}}$ that describes the cost that is expected to accumulate following the policy $\pi_{j}$ from the given state. To avoid any confusion, it is important to point out that a policy $\pi_{j}$ is not a particular sequence of actions - it is a function that presents the best action plan (i.e., a sequence of actions $u_{j}$) to take from any state $x_{t}$ to either acquire the goal *j* or to avoid the obstacle *i*-depending on whether the controller is attached to a goal or an obstacle - (i.e., $\pi_{j}$($x_{t}$) = $u_{j}$ = [*u*_1_, *u*_2_, ..., $u_{T_{j}}$]).

Returning to the reaching scenario in Fig 1, the control systems associated with the targets and the obstacle generate policies $\pi_{i}$($x_{t}$), *i* = [1,4]. Each policy is accompanied by a reward or loss and an action cost, whose values may change with time and previous actions. The reward values refer to the expected benefits for reaching a target. The loss values represent the expected cost associated with colliding with an obstacle. Finally, the action cost refers to the cost (i.e., effort) required to implement each policy at a given state. Selecting between alternative policies requires integrating multiple decision variables online, that is, continuously and dynamically while acting. However, these decision variables are expressed in different “currencies”, making their integration into a unified value representation challenging. To address this, we adopt a probabilistic approach to integrate value information into a common currency. At first, the decision variables are compared within three distinct spaces: i) **goods-space**, where the expected reward values associated with individual goals are compared, ii) **obstacle-space**, where the expected loss values resulting from potential collisions are assessed—this space was simplified and integrated into the goods-space in the present study, represented through the collision probability rather than a separately parameterized loss magnitude, iii) **action-space**, where the action costs of executing policies to achieve the goals and avoid obstacles are compared. The outputs from these comparisons are integrated into a scalar value named “relative desirability” and characterize how desirable it is to follow a particular policy with respect to the alternatives at a given time. It takes values between 0 (undesirable) and 1 (highly desirable) and is used to weight the contribution of the individual policies to the mixed-policy that will be followed by the hand at a given state.

### Modeling action selection in dynamic environments

This section briefly presents the computational theory developed to model action-selection tasks in dynamic environments. More details about the model are presented analytically in [17]. We used a simulated reaching experiment as a paradigm, although the framework can be extended to model other tasks, such as grasping.

### Multiple competing goals in obstacle-free environments

The computational model is based on stochastic optimal control theory, which has been extensively used over the past years to model goal-directed movements, such as reaching to touch targets, pointing to a direction and grasping objects [28,50,51]. The basic idea of the optimal control theory is that movement policies π($x_{t}$), at any given state $x_{t}$, are generated by optimizing performance criteria that include both goal- and action-related costs. We have proposed a probabilistic theory [17] to model action-selection tasks with multiple competing goals. According to this theory, the action-selection problem in dynamic environments with competing goals can be decomposed into policy solutions for the individual goals. Consider a reaching scenario with N targets presented at different locations from the current hand state $x_{t}$. What is the best policy that someone should follow so as to maximize the expected reward and simultaneously minimize the effort cost? According to our theory, the best policy $\pi_{mix}$($x_{t}$) can be approximated as a weighted average of the best individual policies $\pi_{j}$($x_{t}$) - i.e., the policies generated to reach each of the individual targets:


$$\begin{array}{c} \pi_{mix}(x_{t})=\sum_{j=1}^{N}v_{j}(x_{t})\pi_{j}(x_{t}) \end{array}$$ (1)

Where $v_{j}$ is the weight of the policy $\pi_{j}$ at the state $x_{t}$ - i.e., how much this individual policy contributes to the overall policy $\pi_{mix}$ followed by the agent at that state.

Each of the individual optimal policies $\pi_{j}$ is generated by the minimization of the cost function in Eq 2, which describes the cost for reaching the target *j* with zero velocity, starting from the current state $x_{t}$ and following the policy $\pi_{j}$ for time instances t = [*t*_1_, *t*_2_, ... $t_{T_{j}}$]:


$$\begin{array}{c} J_{j}^{G}=(x_{T_{j}}-Sp_{j}{)}^{T}Q_{T_{j}}^{G}(x_{T_{j}}-Sp_{j})+\sum_{t=1}^{T_{j}-1}\pi_{j}(x_{t}{)}^{T}R\pi_{j}(x_{t}) \end{array}$$ (2)

Where $T_{j}$ is the number of time steps over which the cost is optimized for reaching target *j*, which is specified per task configuration as a parameter of the optimization problem [28,52], S is a matrix that picks out the hand and the target location from the state vector $x_{t}$. The first term of the cost function $J_{j}$ describes the accuracy cost that penalizes policies that drive the hand away from the location of target *j*. The second term is the motor command cost that penalizes policies with high effort to reach the target *j*. Both the accuracy cost and the motor command cost characterize what we call “action cost” $V_{\pi_{j}}$ for implementing the policy $\pi_{j}$ at the given state. Matrices $Q_{T_{j}}^{G}$ and *R* define the precision- and control- dependent costs, respectively. Given the current state $x_{t}$ and the location of the target *j*, the controller generates the policy $\pi_{j}$ that minimizes the expected cost in Eq 2. This is a modified form of the Linear Quadratic Gaussian (LQG) regulator in optimal control theory, in which the system dynamics are linear, the cost function is quadratic and the noise is Gaussian, but with signal dependent noise [52]. Specifically, the motor commands are subject to signal-dependent noise, in which zero-mean Gaussian noise scales proportionally with command magnitude, while sensory observations are corrupted by additive Gaussian noise applied independently to position and velocity states [52].

### Multiple competing goals in cluttered environments

In natural tasks, we often do not act on isolated targets but in cluttered environments. To successfully reach a target in the presence of nearby obstacles, the hand should deviate away from the obstacles, in order to reach the target without collision. In the current study, we extended the computational theory presented above to model reaching movements in cluttered environments. The first step was to construct a cost function for obstacle-avoidance, similar to the one developed for reaching to targets. According to this cost, the hand should move as far away as possible from the current location of the obstacle, while exerting as little effort as possible. One of the simplest approaches was to use the Euclidean distance between the hand location and the location of the obstacle to define the cost function. However, this cost function is not quadratic and therefore the LQG framework is not directly applicable. Another solution, which has been extensively used by other studies, is to force the hand to pass through a via-point located near the obstacle [53,54]. However, this approach biases action-selection towards particular pre-defined locations, and therefore it is not consistent with human experimental studies revealing high variability in trajectories for obstacle avoidance [7,30].

In the current study, we proposed an alternative approach to construct an approximate policy for avoiding an obstacle at a given state. Instead of computing directly the policy for avoiding the obstacle, we computed the policy π($x_{t}$) to reach the obstacle from the current state without velocity constraints at the end of the movement, and then “negate” that policy. Hence, the cost function for avoiding the obstacle *k* is given as:


$$\begin{array}{c} J_{k}^{O}=(x_{T_{k}}-Sp_{k}{)}^{T}Q_{T_{k}}^{O}(x_{T_{k}}-Sp_{k})+\sum_{t=1}^{T_{k}}\pi_{k}(x_{t}{)}^{T}R\pi_{k}(x_{t}) \end{array}$$ (3)

The minimization of Eq. 3 provides the optimal policy $\pi_{k}$($x_{t}$) to reach the obstacle *k* without any velocity constraints. By negating this policy, we get an approximate policy to avoid the obstacle. The time $T_{k}$ is the time to contact the obstacle *k*. Similar to the cost function of the targets, the first term describes the goal of the controller - i.e., arrive at the obstacle - and the second term is the effort required to arrive at the obstacle.

When multiple targets and obstacles are presented simultaneously on the field, the framework follows a mixture of policies $\pi_{mix}$, which is given as a weighted average of the individual policies related to the targets (i.e., goals) $\pi^{G}(x_{t})$ and the obstacles $\pi^{O}(x_{t})$ at any given state $x_{t}$:


$$\begin{array}{c} \pi_{mix}(x_{t})=\sum_{j=1}^{N}v_{j}^{G}(x_{t})\pi_{j}^{G}(x_{t})+\sum_{k=1}^{M}v_{k}^{O}(x_{t})\pi_{k}^{O}(x_{t}) \end{array}$$ (4)

Where $v_{j}^{G}(x_{t})$ and $v_{k}^{O}(x_{t})$ describe the contribution (i.e., weights) of each of the target-policy *j* and obstacle-policy *k*, respectively, to the mixed policy $\pi_{mix}$ followed by the model at the given state $x_{t}$. Note that the computational framework forms a weighted combination of competing policies and executes only part of this control sequence before re-evaluating the next mixture of policies (i.e., see S5 Text in Christopoulos et al. [17] for a discussion of “receding horizon control theory”).

So far, we assumed that both obstacles and targets have the same influence on the mixture of the individual policies. However, experimental studies have shown individual differences in risk aversion in both humans and animals. For instance, when rushing to catch a falling object, a person might reach more directly and accept higher collision risk with nearby items, whereas a casual reach for the same object would follow a more cautious, obstacle-avoiding trajectory. To take the different levels of risk aversion into account, we introduce a scalar variable α that determines the risk aversion of the model. It takes values between 0 and 1, such that when α is close to 0, the model focuses mostly on avoiding the obstacles, and when α is close to 1 the model pays more attention to reaching the targets. Given the level of risk aversion, we write Eq 4 as:


$$\begin{array}{c} \pi_{mix}(x_{t})=\sum_{j=1}^{N}\frac{\alpha v_{j}^{G}(x_{t})}{\alpha \sum_{j=1}^{N}v_{j}^{G}(x_{t})+(1-\alpha )\sum_{k=1}^{M}v_{k}^{O}(x_{t})}\pi_{j}^{G}(x_{t})+\sum_{k=1}^{M}\frac{\left(1-\alpha )v_{k}^{O}(x_{t}\right)}{\alpha \sum_{j=1}^{N}v_{j}^{G}(x_{t})+(1-\alpha )\sum_{k=1}^{M}v_{k}^{O}(x_{t})}\pi_{k}^{O}(x_{t}) \end{array}$$ (5)

Where N and M are the number of targets and obstacles, respectively.

#### Computing target and obstacle policy desirability.

The remaining problem is to compute the weighting factors for the targets, $v^{G}(x_{t})$, and the obstacles, $v^{O}(x_{t})$. Consider the simplest scenario introduced in our previous work [17], in which reaches are performed in an obstacle-free environment. In this scenario, $\pi_{mix}(x_{t})$ is given as a weighted average of the individual policies $\pi_{j}(x_{t})$ related to each of the targets *j*. The weights $v^{G}(x_{t})$ are the relative desirability values defined above, reflecting how “desirable” it is to follow a particular policy $\pi_{j}(x_{t})$ (in this case, reach to the target *j*) with respect to the alternative options, and are given as:


$$\begin{array}{c} v_{j}^{G}(x_{t})=P(V_{\pi_{j}}(x_{t})<V_{\pi_{i\neq j}}(x_{t}))P(r_{j}>r_{i\neq j}) \end{array}$$ (6)

The first term is the “action-related” component of the relative desirability $v_{j}^{G}(x_{t})$ and describes the probability that reaching for the target *j* has the lowest cost (i.e., requires less effort) compared to the rest of the alternatives, at the given state $x_{t}$. The second term is the “goods-related” component and describes the probability that selecting the target *j* will have the highest outcome (i.e., reward) compared to the alternatives, at that state. The first component of the relative desirability can be approximated by the soft-max transformation of the policy cost $V_{\pi_{j}}(x_{t})$ at the current state, as:


$$\begin{array}{c} P(V_{\pi_{j}}(x_{t})<V_{\pi_{i\neq j}}(x_{t}))=\frac{\lambda e^{-\frac{1}{\lambda }V_{\pi_{j}}(x_{t})}}{\sum_{i=1}^{N}\lambda e^{-\frac{1}{\lambda }V_{\pi_{i}}(x_{t})}} \end{array}$$ (7)

Where λ is the “free inverse temperature” parameter.

The second component of the relative desirability is determined by the expected benefits related to the alternative options. Our previous work [17] explored this component in simplified contexts. For instance, in a simple case where the amount of reward is fixed and equal for all goals, the relative desirability for a policy $\pi_{j}$ was modeled as the probability of receiving the reward for a given target *j*:


$$\begin{array}{c} P(r_{j}>r_{i\neq j})=\rho_{j} \end{array}$$ (8)

However, this model does not account for risks, such as those encountered when reaching for objects in a cluttered environment. In such scenarios, evaluating the “goods-related” component becomes more complex, as the potential reward for success must be weighed against the potential penalty for colliding with obstacles. In the present simulations, the target reward is held constant for simplicity. To address this challenge, we propose a new formulation for the “goods-related” component based on the probability of collision with obstacles.

To compute this collision probability, we defined an expanded collision zone surrounding each obstacle, characterized by two concentric circles: an inner circle with diameter $d_{inner}$ equal to the physical size of the obstacle, and an outer circle with diameter $d_{outer}$ that delineates the extended region where collision risk is perceived. The collision cone is formed by computing tangent lines from the current hand position to both the inner and outer circles, creating a spatial region that extends from the hand toward and around the obstacle. For a given hand position at state $x_{t}$, collision probability is computed based on its location relative to these boundaries. Points within the inner circle are assigned a collision probability of 1, while points between the inner and outer circles exhibit linearly decreasing probability:


$$\begin{array}{c} P_{position}(d)=\left\{ \begin{array}{cc} 1, & d\leq d_{inner}\\ 1-\frac{d-d_{inner}}{d_{outer}-d_{inner}}, & d_{inner}<d<d_{outer}\\ 0, & d\geq d_{outer} \end{array}\right. \end{array}$$ (9)

where *d* is the diameter of the circle, centered on the obstacle, that passes through the current hand position. To evaluate collision risk along a trajectory, we define an indicator function that incorporates both spatial location and movement direction:


$$\begin{array}{c} I(x_{t})=\left\{ \begin{array}{cc} P_{position}(d), & {\dot{x}}_{t}\text{ within cone}\\ 0, & \text{otherwise} \end{array}\right. \end{array}$$ (10)

where ${\dot{x}}_{t}$ is the velocity vector at state $x_{t}$. Over a trajectory spanning *H* future time steps, the overall collision probability is then computed as:


$$\begin{array}{c} P(\text{collision}|x_{t})\propto \frac{\sum_{\tau =t}^{t+H}I(x_{\tau })}{H} \end{array}$$ (11)

Where the value of $d_{outer}$ was set to reflect the spatial scale of the simulated task and the physical size of the obstacle. In the model, this parameter defines a tolerance boundary used in the computation of collision probability and effectively represents a safety buffer around the obstacle. The value was selected for computational convenience and was not derived from experimental measurements of human clearance distances. Importantly, the framework does not require this boundary to be fixed. Empirically measured safety margins or obstacle-dependent tolerance boundaries could be incorporated without modifying the remainder of the model. The implications of this simplification are discussed in the Discussion section.

Therefore, the “goods-related” component of the relative desirability $v_{j}^{G}(x_{t})$ is approximated by the probability of *NOT* colliding with the obstacle (the probability of going to the target), which is defined by:


$$\begin{array}{c} P(\text{target}|x_{t})=1-P(\text{collision}|x_{t}) \end{array}$$ (12)

Similarly, the relative desirability for avoiding the obstacle *k* is given as:


$$\begin{array}{c} v_{k}^{O}(x_{t})=P(V_{\pi_{k}}(x_{t})<V_{\pi_{i\neq k}}(x_{t}))P(r_{k}>r_{i\neq k}) \end{array}$$ (13)

Where the first term is the action-related component of the relative desirability $v_{k}^{O}(x_{t})$, which describes the probability that avoiding the obstacle *k* has the lowest cost compared to the alternative actions, at the current state, and could be approximated by:


$$\begin{array}{c} P(V_{\pi_{k}}(x_{t})<V_{\pi_{i\neq k}}(x_{t}))=\frac{\lambda e^{-\frac{1}{\lambda }V_{\pi_{k}}(x_{t})}}{\sum_{i=1}^{M}\lambda e^{-\frac{1}{\lambda }V_{\pi_{i}}(x_{t})}} \end{array}$$ (14)

And the second term is the goods-related component of the relative desirability $v_{k}^{O}(x_{t})$, and could be simply defined by $P(\text{collision}|x_{t})$.

## Supporting information

S1 Table Model parameters. The values of the model parameters used in the simulations.
(XLSX)

S1 Fig Simulated reaching trajectories using action cost-driven relative desirability. Simulated reaching trajectories (green lines) for a large obstacle (diameter = 4) positioned along the direct path to the target, using the same spatial configuration as Fig 4D. In this simulation, relative desirability is computed using only the action-related component, without incorporating collision probability. The origin is indicated by a black dot, the target by a blue dot, and the obstacle by a red circle.
(TIFF)

S2 Fig Simulated reaching trajectories with alternative collision probability decay functions. Simulated reaching trajectories (green lines) for three obstacle sizes using two alternative decay functions: exponential decay (A–C) and Gaussian CDF-based decay (D-F). (A, D) Small obstacle (diameter = 1), (B, E) medium obstacle (diameter = 2), (C, F) large obstacle (diameter = 4). The origin is indicated by a black dot, the target by a blue dot, and obstacles by red circles.
(TIFF)
