# Supplementary material for: An integrative computational approach for obstacle avoidance during action selection
Source: PLoS Comput Biol. 2026 Oct 26;22(10):e1013756. doi: 10.1371/journal.pcbi.1013756 (PMC13719039; doi:10.1371/journal.pcbi.1013756)
Supplement: Supplementary file 1 [file pcbi.1013756.s006.pdf]

Reviewer 1

Major Comments

(Line ~205)

The selection of the tolerance boundary (outer diameter of the expanded collision zone) appears fixed and somewhat arbitrary. While the approach is computationally convenient, prior experimental work suggests that safety margins are not constant but instead vary systematically with obstacle geometry and material properties, reflecting differences in perceived collision risk.

Experimental studies quantifying minimum clearance distance during 3D reaching tasks have shown that clearance depends on obstacle shape, size, and fragility, and that perceived risk can be formalized using probabilistic or Bayesian decision-theoretic frameworks, like Tresilian (1998): Attention in action or obstruction of movement? A kinematic analysis of avoidance behavior in prehension; Trommershäuser et al. (2008): Decision making, movement planning and statistical decision theory; Yang et al. (2021a): A collision avoidance algorithm for human motion prediction based on perceived risk of collision – Part 1: Model development; Yang et al. (2021b): A collision avoidance algorithm for human motion prediction based on perceived risk of collision – Part 2: Application; Baus & Yang (2025): Minimum clearance distance during upper-extremity collision avoidance reaching tasks incorporating perceived risk.

Discussing such findings would help contextualize the choice of tolerance boundaries in the current model and clarify whether the fixed buffer represents a simplification or a principled approximation. At minimum, the authors should explicitly justify the choice of the tolerance boundary and discuss how obstacle-dependent or experimentally informed buffer zones could be incorporated in future extensions.

We thank the reviewer for this constructive comment and for directing us to the relevant literature on perceived risk and clearance distance. We agree that the manuscript did not sufficiently justify the choice of the tolerance boundary or contextualize it within the broader empirical literature on obstacle avoidance behavior. The outer boundary parameter in our model was chosen to reflect the spatial scale of the simulated task and the physical size of the obstacle for computational simplicity, and is not derived from empirical measurements of clearance distance or perceived risk. We acknowledge this as a simplification. Importantly, the present model is developed through computational analysis and was not fit to experimental reaching data. As such, the tolerance boundary should be viewed as a configurable parameter rather than a fixed physiological quantity. Within our framework, the tolerance boundary enters only in the computation of collision probability, while the relative desirability framework that integrates collision probability with action costs is independent of how this boundary is defined. Consequently, the current fixed boundary can be readily replaced by an empirically informed, obstacle-dependent buffer derived from behavioral measurements of safety margins. In this sense, the boundary does not necessarily represent the geometric extent of the obstacle itself, but rather the effective safety distance required to avoid collision, which could be modulated based on obstacle properties (e.g., size, shape, fragility) or experimentally measured clearance distances. We have added a brief justification of the boundary choice to the Methods section and expanded the Limitations section to discuss more thoroughly, including directions for future extensions. For the relevant changes, see lines 357-372 and lines 585-593 in the revised manuscript.

(Line ~208)

The linear decay of collision probability between the inner and outer boundaries is intuitive. Still, it is unclear whether this assumption is supported by empirical or theoretical work, or whether it was selected primarily for simplicity. Prior studies on risk-sensitive motor control and collision avoidance suggest that perceived risk may scale nonlinearly with distance and uncertainty.

The authors are encouraged to clarify whether alternative decay functions were considered and to discuss how sensitive the model behavior is to this assumption. Even a brief justification or acknowledgment of this limitation would strengthen the modeling rationale.

Thank you for raising this point. The linear decay was selected primarily for simplicity and analytical transparency, and we acknowledge that it is not derived from empirical or theoretical principles regarding perceived risk scaling. To assess whether this choice materially affects the model's qualitative predictions, we repeated the simulations corresponding to Fig. 4B–D using two alternative decay functions: an exponential decay function (where $k = 3$) and a Gaussian CDF-based decay function (where $\sigma = 0.33$), where all other parameters were consistent with the simulations with linear decay. The resulting trajectories are shown in Supplementary Figure S2. Across all three decay functions, the simulated reaching behaviors are qualitatively indistinguishable, indicating that the specific form of the decay function is not a primary driver of the simulated reaching patterns reported in this study. This robustness is expected because the three decay functions share the same boundary conditions, with collision probability equal to 1 at the obstacle surface and 0 at the tolerance boundary and differ only in their intermediate profiles. Because the qualitative trajectory is shaped by the cumulative effect of policy weighting over many time steps, moderate local differences in the decay gradient do not alter the overall reaching pattern. We note that this comparison is not intended as a systematic parameter sensitivity analysis, nor were the parameters of the alternative functions optimized, since such an analysis falls outside the scope of the present theoretical framework. The comparison demonstrates that the qualitative predictions of the model are robust to reasonable variation in the decay function. We have added a brief discussion of this point to the manuscript (see lines 246-252).

Results and Discussion:

Throughout the Results and Discussion, the manuscript states that the model behavior is "consistent with human studies." While this appears qualitatively correct, the validation remains entirely qualitative. This is acceptable, but it should be clearly framed as such.

The authors may consider explicitly acknowledging this limitation and briefly discussing whether simple quantitative metrics (e.g., trajectory curvature, clearance distance, or deviation magnitude) could be extracted for future comparison with experimental data.

We appreciate the reviewer for pointing this out. We agree that the qualitative nature of the comparisons with human studies should be stated more explicitly and clearly. Accordingly, we have revised the opening sentence of subsection 2.3 of the Results section to more clearly frame the comparisons as an evaluation of whether the model reproduces characteristic patterns of human behavior, rather than a quantitative validation (see lines 231-233). Importantly, the proposed model is a system-level framework designed to qualitatively predict motor behavior in action regulation tasks. As such, it focuses on the interaction between cognitive and decision-related processes that shape behavioral trajectories, rather than on the detailed biomechanics of limb movements. Human movement trajectories also reflect biomechanical constraints (e.g., limb dynamics and musculoskeletal limitations), which are not explicitly incorporated in the present model. In addition, we have expanded the *Limitations and future perspectives* section to identify specific quantitative metrics, which could serve as benchmarks for future systematic comparisons with human experimental data (see lines 384-391). We believe these revisions clarify the scope of the present study while pointing toward a concrete path for quantitative benchmarking in future work.

(Line ~530)

The current collision probability model assumes obstacles can be represented using concentric circular boundaries. While this is reasonable for proof-of-concept simulations, real-world obstacles often have irregular or anisotropic geometries.

Recent experimental work using full 3D obstacle surfaces and segment-level clearance metrics suggests that obstacle shape plays a critical role in avoidance strategies (e.g., Chapman & Goodale, 2008: Missing in action: the effect of obstacle position and size on avoidance while reaching; Baus & Yang, 2025). A brief discussion of how the framework might be generalized to irregular obstacle geometries would improve the model's scope and realism.

Thank you for raising this point. Empirical work on human reaching has shown that avoidance behavior is sensitive to specific geometric properties of obstacles, including their height (Chapman & Goodale, 2008), spatial extent and orientation (Dean & Brüwer, 1994), and three-dimensional shape, material, and orientation (Baus & Yang, 2025). The circular boundary assumption in this study is indeed a deliberate simplification, and the underlying framework does not depend on this simplification. Specifically, the circular assumption resides in the collision probability computation, where collision cones are constructed via tangent lines to concentric circles. The relative desirability framework that integrates collision probability with action costs operates independently of how the collision probability itself is computed. The collision cone formalism has been generalized to objects of arbitrary shape (Chakravarthy & Ghose, 1998), providing a theoretical basis for extending the directional collision check beyond circular boundaries. Because this generalization reduces an irregular obstacle to an equivalent enclosing cone, it could be integrated with the current model's graded probability computation, which assigns collision probability based on membership within a hierarchy of nested cones. Developing the full implementation for irregular geometries remains a direction for future work. We have added a discussion of this point to the Limitations and Future Perspectives section (see lines 357-372).

Minor Comments:

- In Figure 2 and several subsequent figures, axes lack labels and units. Please add axis names and units for clarity.

Thank you for raising this concern. We have updated figures 2, 4, 5 and 6 to add axes labels and units. The probability of collision and relative desirability values are unitless.

- Additionally, please explain why axis limits differ across panels, as this may affect visual comparison.

Thank you for pointing this out. In the previous version, there were inconsistencies in the size of the displayed maps. We have revised figures 2 and 3 to unify the axis limits.

- Although placing Methods after the Discussion follows PLOS formatting conventions, readers may benefit from earlier access to key modeling details, particularly to aid interpretation of simulated trajectories.

Thank you for this important concern. We agree that early access to key modeling details aids interpretation of the simulated trajectories, and we have addressed this within the constraints of PLOS formatting conventions, which require the Methods section to follow the Discussion. Specifically, we have added pseudocode (Algorithm 1, "Simulated Trajectory Generation") at the beginning of the Results subsection, immediately before figure 4 was presented. The algorithm provides a conceptual overview of the closed-loop simulation procedure, including the computation of control policies, the per-step mixing of motor commands by relative desirability, and the iterative update of collision probability, without requiring prior exposure to the mathematical equations developed in the Methods section. This allows readers to follow the logic of the simulations and interpret the resulting trajectories in context, while the full technical details remain accessible in the Methods section.

- Line ~436: missing comma.

We thank the reviewer for this comment. We have added a colon to this line (new line 478) for completeness.

References

1. Chapman CS, Goodale MA. Missing in action: the effect of obstacle position and size on avoidance while reaching. Experimental Brain Research. 2008 Oct;191(1):83-97.
2. Dean J, Brüwer M. Control of human arm movements in two dimensions: paths and joint control in avoiding simple linear obstacles. Experimental brain research. 1994 Jan;97(3):497-514.
3. Baus J, Yang J. Motion prediction and validation considering perceived risk-based three-dimensional collision avoidance. Proceedings of the Institution of Mechanical Engineers, Part H: Journal of Engineering in Medicine. 2025 Aug;239(8):802-14.
4. Chakravarthy A, Ghose D. Obstacle avoidance in a dynamic environment: A collision cone approach. IEEE Transactions on Systems, Man, and Cybernetics-Part A: Systems and Humans. 2002 Aug 6;28(5):562-74.

Reviewer 2

This manuscript presents a computational model for action selection in cluttered environments, extending the relative desirability framework (Christopoulos & Schrater, 2015) to incorporate obstacle avoidance. The authors demonstrate through simulations that the model captures qualitative features of human reaching behavior, including trajectory deviations around obstacles and the transient tendency to initiate movements toward obstacles before avoidance.

The paper addresses an important problem in motor control and presents a theoretically interesting framework. The extension of relative desirability to obstacle avoidance is novel, and the biological plausibility argument is well-articulated. However, addressing the concerns outlined below would strengthen the manuscript and improve its suitability for publication.

General comments:

1. The paper relies entirely on qualitative comparisons with previously published human studies. The manuscript would be significantly strengthened by quantitative comparisons with human behavioral data from cited studies using statistical metrics comparing model predictions to human performance.

Thank you for this insightful comment. We agree that quantitative comparison with human behavioral data is an important direction. However, we respectfully note that such analysis falls outside the scope of the present work. The computational framework introduced here operates at a systems level and is intended to provide a theoretical account of the computational principles underlying action selection in cluttered environments, rather than to reproduce specific experimental datasets. The model parameters are not calibrated to match the specific experimental conditions of any individual study. In this regard, our approach is consistent with a broader line of work in computational motor control that first establishes theoretical principles and demonstrates qualitative behavioral predictions, prior to quantitative benchmarking. This includes influential frameworks such as the affordance competition hypothesis (Cisek, 2007) and the relative desirability framework (Christopoulos & Schrater, 2015), which the present work extends.

Importantly, this study follows the same modeling philosophy as our previous studies published in PLOS Computational Biology, where we developed systems-level models capable of *qualitatively* predicting motor behavior (Christopoulos, et al., 2015, Zhong et al., 2024, Zhong et al., 2025). As in those studies, the current model incorporates simplifying assumptions that are standard in control theory. For example, the dynamics of the hand are approximated as a point mass, a common approach that enables tractable modeling of control policies but does not capture the full biomechanical constraints of the limb. As a result, the model is not intended to generate precise quantitative predictions of human performance.

A meaningful quantitative comparison with human data would require re-parameterizing the model to match the specific conditions of a given experiment, including details such as movement kinematics, obstacle geometry, and task constraints. Such an effort would constitute a separate line of investigation beyond the goals of the present theoretical framework. We do agree that the qualitative nature of our comparisons and the limitations of the model should be more clearly articulated, as well as the path toward future quantitative validation. We have revised the manuscript accordingly (see lines 384-391).

2. The model's ability to produce trajectories that initially deviate toward obstacles (Figure 5) is presented as a key success. It is unclear how the policy mixture produces initial motion toward the obstacle. This is a critical gap because this phenomenon is highlighted as evidence that the model captures human behavior.

Thank you for raising this question. The occasional toward-obstacle trajectories arise from the interaction between two features of the model: the temporal structure of collision probability feedback, as well as the presence of control-dependent and signal-dependent noise (Todorov & Jordan, 2002). At movement onset, the hand has not yet approached the obstacle, so collision probability is low and the policy mixture is dominated by the target-approach policy. Because motor commands are corrupted by signal-dependent noise, the initial movement direction is variable across trials. In a subset of trials where noise displaces the initial steps toward the obstacle side, the hand enters the collision zone, collision probability rises, the avoidance policy gains weight in the mixture, and the trajectory subsequently curves to go beyond the obstacle before turning back to reach the target. This trial-variable occurrence is consistent with previous literature on human reaching in the presence of non-target objects. Welsh and Elliott (2004) demonstrated that trajectory deviations in the presence of competing stimuli depend on the activation levels of competing motor responses at the moment of movement initiation. When inhibition of a competing response has not resolved by movement onset, the trajectory deviates toward the non-target, and when inhibition has had sufficient time to develop, the trajectory deviates away. At a broader theoretical level, this behavior is also consistent with the minimal intervention principle of optimal feedback control (Todorov & Jordan, 2002): corrections are applied only to deviations that are task-relevant. Liu and Todorov (2007) provided experimental support for this principle in obstacle avoidance reaching, showing that hand-path variability near an obstacle does not decrease in the obstacle vicinity, indicating that the controller does not enforce a precision requirement at intermediate locations. Our model shares the same logic, where avoidance response weight increases only once the trajectory enters the collision zone, leaving earlier deviations uncorrected. We have added a brief mechanistic explanation of this effect in the Results section (see lines 259-267) and expanded the discussion of its theoretical implications in the Discussion section (see lines 320-327).

3. No algorithm or pseudocode explains how simulations were performed. Readers cannot understand or reproduce the trajectory generation process.

Thank you for raising this concern. We agree that the original manuscript lacked an explicit description of the simulation procedure, and we have addressed this in the updated manuscript. We have added Algorithm 1 ("Simulated Trajectory Generation") in the Results section immediately before figure 4 was presented. The algorithm describes the complete closed-loop simulation procedure, including: (1) the computation of optimal control policies for target approach and obstacle avoidance; (2) the per-step generation of competing motor commands; (3) the evaluation of effort costs and collision probability at each state; (4) the computation of relative desirability and the mixing of commands weighted by desirability and the risk aversion factor α; (5) the application of signal-dependent motor noise; and (6) the iterative update of collision probability from the executed trajectory, which feeds back into the next step. The simulation loop runs until the hand reaches the target, reproducing the closed-loop nature of the process. The algorithm provides a conceptual overview of the closed-loop simulation procedure without requiring prior exposure to the mathematical equations developed in the Methods section. This allows readers to follow the logic of the simulations and interpret the resulting trajectories in context, while the full technical details remain accessible in the Methods section. In addition, all key simulation parameter values are reported in the supplementary materials, and the simulation code will be made available on Github upon manuscript publication to support full reproducibility.

Additional suggestions:

1. The "75% completion" criterion for velocity magnitude (line 174) needs justification.

Thank you for raising this point. To clarify, the 75% completion criterion is used solely for visualization. We used this value to define a representative velocity magnitude when projecting the position-velocity desirability space onto

two dimensions for display purposes. This approach follows the method introduced in Christopoulos and Schrater (2015), where the analogous value of 85% was used for the same purpose. Neither value carries particular significance, and any reasonable choice in this range would yield qualitatively identical maps. We have added a brief clarification in the manuscript (see lines 175-177).

2. Figures 2-3: Velocity arrows are invisible at normal viewing size. Either increase arrow size or add an inset showing magnified arrows with explanation.

Thank you for this suggestion. We have added insets showing magnified arrows in all subfigures in figures 2 and 3, with added explanation in the corresponding figure legends.

3. Figure 4: Obstacle sizes described as "small," "medium," "large" should be specified numerically

Thank you for pointing this out. We have updated figure 4 to describe the obstacles as “small obstacle (diameter = 1)”, “medium obstacle (diameter = 2)”, and “large obstacle (diameter = 4)” to improve clarity. We have also added units for the x and y axes for figures 2-6 (a.u. for x and y position).

4. How was movement duration T determined for each simulation? Is it fixed or computed dynamically?

Thank you for this question. In the manuscript, T denotes the time window over which the cost function is optimized and the optimal gain matrices are computed. The LQG formulation used here follows the approach in (Todorov and Jordan, 2002; Todorov, 2005), where the cost is optimized over a fixed number of time steps. The value of T is set per task configuration and does not carry strict physical significance in the simulations within the scope of this study, as the systems-level model is not designed for quantitative comparison with human movement timing data. Importantly, the “actual” movement duration in each simulation is not prescribed by T but emerges dynamically from the execution process. The model operates through an iterative replanning loop, where in each iteration, the initial part of the optimal trajectory is executed, the state is updated, and the collision probability is recomputed at the new state. This loop continues until the Euclidean distance between the simulated hand position and the target falls below a predefined contact threshold, at which point the movement is considered complete. Consequently, different task configurations produce different total movement durations without any manual adjustment. This iterative replanning strategy is conceptually equivalent to receding horizon control theory, where the optimal control policy is repeatedly recomputed over a finite horizon while only a portion of the control sequence is executed before re-optimization. We have added a clarification in the methods section of the manuscript (see lines 479-481 and lines 528-531).

5. The "rim" artifact in Figure 2D is acknowledged but unresolved. Does this artifact affect any trajectory predictions, and if not, why not?

Thank you for raising this question. The rim artifact visible in Fig. 2D is a consequence of relying solely on action cost to compute relative desirability, and it does affect trajectory predictions when we integrate collision probability into the model. To illustrate this, we have added a supplementary figure (Fig. S1) showing simulated trajectories generated using only cost-based relative desirability. The elevated avoidance desirability near the target causes trajectories to deviate away from the target from the beginning of the trial, or to completely ignore the obstacle and colliding into it, producing qualitatively implausible behavior. Although this illustration is based on one representative parameter configuration, it demonstrates that relying on action cost alone is less likely to generate behaviorally plausible trajectories. Importantly, Figure 2 presents the action cost-driven scenario as an intermediate illustration to motivate the need for incorporating collision probability. All trajectory simulations in the paper (Figs.

4–6) use the comprehensive model, in which collision probability is integrated into the goods-related component of relative desirability, therefore the rim is effectively suppressed. We have added a clarification of this point in the results section of the manuscript (see lines 199-203).

6. What noise model was used to generate trial-to-trial variability? What are the noise parameters?

Thank you for pointing this out. The model generates trial-to-trial variability through two noise sources implemented within the LQG framework based on Todorov(2005)'s work. Trial-to-trial variability is generated by signal-dependent, or multiplicative motor noise, in which zero-mean Gaussian noise is added to the motor command at each time step with standard deviation scaling proportionally with command magnitude. Sensory observations are additionally corrupted by additive Gaussian observation noise, with independent noise applied to position and velocity estimates. We added a description of this noise model to the Methods section (see below). All noise parameters, as well as the full set of key model parameters, are reported in the Supplementary Material submitted with the original manuscript. We also added a description of the noise model directly to the Methods section (see lines 492-495).

References

1. Cisek P. Cortical mechanisms of action selection: the affordance competition hypothesis. Philosophical Transactions of the Royal Society B: Biological Sciences. 2007 Sep 29;362(1485):1585-99.
2. Christopoulos V, Schrater PR. Dynamic integration of value information into a common probability currency as a theory for flexible decision making. PLoS computational biology. 2015 Sep 22;11(9):e1004402.
3. Christopoulos V, Bonaiuto J, Andersen RA. A biologically plausible computational theory for value integration and action selection in decisions with competing alternatives. PLoS computational biology. 2015 Mar 24;11(3):e1004104.
4. Zhong S, Choi JW, Hashoush NG, Babayan D, Malekmohammadi M, Pouratian N, Christopoulos V. A neurocomputational theory of action regulation predicts motor behavior in neurotypical individuals and patients with Parkinson's disease. PLOS Computational Biology. 2022 Nov 17;18(11):e1010111.
5. Zhong S, Pouratian N, Christopoulos V. Computational mechanism underlying switching of motor actions. PLoS Computational Biology. 2025 Feb 10;21(2):e1012811.
6. Todorov E, Jordan MI. Optimal feedback control as a theory of motor coordination. Nature neuroscience. 2002 Nov 1;5(11):1226-35.
7. Welsh TN, Elliott D. Movement trajectories in the presence of a distracting stimulus: Evidence for a response activation model of selective reaching. The Quarterly Journal of Experimental Psychology Section A. 2004 Jan 1;57(6):1031-57.
8. Liu D, Todorov E. Evidence for the flexible sensorimotor strategies predicted by optimal feedback control. Journal of Neuroscience. 2007 Aug 29;27(35):9354-68.
