# Supplementary material for: An integrative computational approach for obstacle avoidance during action selection
Source: PLoS Comput Biol. 2026 Oct 26;22(10):e1013756. doi: 10.1371/journal.pcbi.1013756 (PMC13719039; doi:10.1371/journal.pcbi.1013756)
Supplement: Supplementary file 2 [file pcbi.1013756.s007.pdf]

Reviewer #4: The manuscript by Zhong et al. presents a computational framework for obstacle avoidance during action selection that extends a relative-desirability model of multi-target choice to cluttered reaching environments. The authors propose that target-approach and obstacle-avoidance policies are generated in parallel by stochastic optimal controllers and then combined according to a common probabilistic currency, termed relative desirability. Through simulations, the model reproduces several qualitative features reported in human reaching behavior, including trajectory deviations as a function of obstacle position and size, as well as transient initial movements toward an obstacle before later avoidance. The work is conceptually interesting and offers a biologically motivated perspective on how approach and avoidance tendencies may be dynamically integrated during movement planning.

Major points:

- The manuscript is well motivated and conceptually appealing, but the validation is largely qualitative. The model would be substantially strengthened by direct quantitative comparison to human behavioral data and/or formal comparison with alternative computational accounts.

We thank the reviewer for this thoughtful assessment and agree that quantitative comparison with human behavioral data is an important direction for future work. However, the primary objective of the present study is to establish **a systems-level computational framework** capable of reproducing characteristic patterns of obstacle-avoidance behavior and generating mechanistic hypotheses regarding the computations that underlie action selection in cluttered environments, rather than to quantitatively fit data from a specific behavioral experiment. Accordingly, the model was not designed or parameterized to reproduce the conditions of any individual behavioral study. Furthermore, the hand dynamics are approximated as a point mass, a standard simplification in control-theoretic models (e.g., stochastic optimal control models for biological movements) that enables tractable derivation of control policies but does not capture the full biomechanical constraints of the limb. **For these reasons, the present framework is intended to provide qualitative and mechanistic insight into obstacle-avoidance behavior rather than precise quantitative predictions of human performance.**

In this regard, our approach follows a common progression in computational neuroscience, where theoretical frameworks are first developed and evaluated based on their ability to reproduce characteristic behavioral phenomena before being quantitatively benchmarked against experimental datasets. Typical examples include the affordance competition hypothesis (Cisek, 2007), the relative desirability framework (Christopoulos & Schrater, 2015) that the present study extends, and our previous systems-level models published in PLOS Computational Biology (Christopoulos et al., 2015; Zhong et al., 2025). A meaningful quantitative comparison would require re-parameterizing the model to the specific kinematics, obstacle geometry, and task constraints of a particular experiment, which would constitute a distinct line of investigation beyond the goals of the current study. Consistent with this objective, the manuscript evaluates whether the model reproduces characteristic patterns of human behavior and identifies specific behavioral metrics, including collision rate, maximum lateral deviation, initial deviation angle, minimum clearance, and trajectory curvature, that could serve as benchmarks for such future quantitative evaluation (please see lines 429-438).

Regarding alternative computational accounts, we note that the manuscript already contains a formal model comparison. In the Supplementary Material (Figure S1), we compare the full model against a reduced alternative formulation in which relative desirability is computed from the action-related component. This reduced model produces early deviations away from the target and, in some cases, collisions, thereby isolating the specific contribution of the collision-probability term to the generation of behaviorally plausible trajectories. This analysis demonstrates that the principal behavioral signatures of the model depend critically on the interaction between action desirability and collision probability, thereby providing a mechanistic comparison between competing formulations.

With respect to established engineering approaches such as artificial potential fields (APFs), reinforcement learning (RL), and model predictive control (MPC) algorithms, we respectfully suggest that a direct performance benchmark would address a different question than the one investigated here. These methods are typically designed to optimize engineering objectives such as collision avoidance, path efficiency, smoothness, or reward maximization. In contrast, the present framework is intended to explain characteristic features of human obstacle-avoidance behavior. A key objective of the model is not to generate optimal trajectories, but rather to reproduce systematic features of human behavior that often deviate from optimality, including transient initial movements toward obstacles, graded avoidance responses, and occasional collisions when avoidance costs are high. In other words, a model that never collides with an obstacle and always generates the shortest or safest trajectory may be optimal from an engineering standpoint, but it does not necessarily provide the best account of human behavior. Humans frequently exhibit systematic departures from optimality, including occasional errors and collisions. The ability of the present model to reproduce such behaviors is therefore not a limitation, but rather a consequence of its goal to capture biologically plausible decision-making and action-selection processes. We addressed this point on the revision (please see lines 380-390).

More broadly, the primary contribution of the present framework is not merely to reproduce behavioral trajectories, but to generate mechanistic hypotheses regarding the computations that underlie obstacle-avoidance behavior. Specifically, the model proposes that action selection emerges from a competition between goal-directed actions (e.g., reaching the target) and obstacle-avoidance actions, each associated with distinct desirability signals that evolve over time. Such hypotheses cannot be evaluated solely through behavioral comparisons and ultimately require neurophysiological testing. We therefore view the present work as a theoretical framework that generates experimentally testable predictions regarding the neural mechanisms of obstacle avoidance, rather than as a model intended primarily for quantitative behavioral fitting.

Finally, we agree that the qualitative nature of the current evidence should be reflected more explicitly in the manuscript. Accordingly, we have revised several statements throughout the text to avoid implying qualitative correspondence, replacing terms such as “predicts” and “closely mirror” with formulations such as “reproduces” and “is qualitatively consistent with” (highlighted in blue in the revised manuscript). We believe these revisions further clarify the scope of the present study while pointing toward a concrete path for quantitative benchmarking in future work.

- The central “policy negation” step is clearly acknowledged as an approximation, but its biological plausibility remains somewhat speculative. This aspect would benefit from deeper mechanistic justification or more cautious framing.

We thank the reviewer for this comment. As the manuscript notes, policy negation is a computational approximation because stochastic optimal control requires a well-defined endpoint, whereas obstacle avoidance has none. We would add, however, that the operation of reversing the sign of a movement-related directional signal has neural precedents in both anti-saccade (Munoz & Everling, 2004) and anti-reach (Gail & Andersen, 2006; Gail et al., 2009) tasks. Furthermore, obstacle avoidance during reaching engages premotor processes distinct from those supporting direct reaching (Pearce & Moran, 2012; Moll & Kuypers, 1977), consistent with the curved, obstacle-circumventing trajectories generated by our model. Nevertheless, we do not claim that the brain literally inverts a control policy. The cited studies involve discrete movements toward a defined or opposite endpoint, whereas our model inverts a continuous policy in the absence of any endpoint. Thus, policy negation should not be interpreted as a direct claim about the neural implementation of obstacle avoidance. Instead, it serves as a computational abstraction inspired by known neural operations that transform movement-related directional representations, while remaining agnostic about the precise circuit-level mechanisms involved.

More broadly, the purpose of the present framework is not to identify the precise neural implementation of obstacle avoidance, but rather to generate mechanistic hypotheses regarding the computations that may underlie action selection in cluttered environments. In this context, policy negation serves as a tractable computational tool for formalizing how obstacle-related influences can compete with goal-directed actions during movement planning. The resulting framework generates experimentally testable predictions regarding the interaction between reaching and avoidance processes, predictions that ultimately require validation through neurophysiological recordings and neural perturbation experiments.

Accordingly, we have framed the policy negation step more cautiously throughout the manuscript and note that a more biologically grounded formulation remains an important direction for future work, potentially building on the affordance competition hypothesis and dynamic neural field accounts of action selection (Cisek, 2007; Christopoulos et al., 2015), as discussed further in the Discussion section. We have revised the “Model Characteristics and Biological Plausibility” subsection (please see lines 352-379).

- Although the paper is framed as integrating rewards, costs, and risks, the implemented goods-related term is simplified largely to collision probability. The manuscript should clarify this reduced scope more explicitly.

We thank the reviewer for this observation and agree that the reduced scope of the goods-related term should be stated more explicitly. Importantly, the present framework is capable of integrating rewards, costs, and risks within a common decision-making architecture. However, the specific implementation examined in this study represents a simplified case tailored to the obstacle-avoidance task considered here. The general formulation of the goods-related term is

inherited from the relative desirability framework of Christopoulos & Schrater (2015), where reward is represented through the comparison of expected outcomes across competing targets. In that formulation, reward plays a critical role because multiple potential goals compete for selection. In the present study, however, movements are directed toward a single target, eliminating competition among alternative goals. As a result, differential reward across targets does not influence action selection and the reward-related component reduces to a constant term. Thus, the reward machinery remains part of the framework, but it is not actively engaged under the task conditions studied here.

With respect to obstacle avoidance, we incorporated obstacle-related influences into the goods-space and represented them through collision probability, such that the goods-related term effectively reduces to the probability of not colliding. This simplification was adopted because the primary objective of the present study was to understand how obstacle-related risk influences action selection, rather than to investigate trade-offs among multiple goals with different reward values. Consequently, the current implementation focuses on the risk component while holding reward constant.

Importantly, this simplification applies only to the implementation used in the present simulations and not to the framework itself. The underlying architecture retains separate reward and loss terms, and obstacle-specific penalties, differential target rewards, or other value-based factors could be incorporated without modifying the remainder of the model. Indeed, the framework was originally developed to account for reward-based competition among multiple potential actions (Christopoulos & Schrater, 2015), and the present work should be viewed as a special case in which reward competition is absent and obstacle-related risk becomes the dominant factor influencing action selection.

To clarify this distinction, we have revised the manuscript where the goods-related term is introduced in the Results (see lines 149-155) and Methods (see lines 485-486). We have also expanded the Limitations and Future Perspectives subsection to explicitly identify differential rewards, obstacle-specific loss magnitudes, and multi-target decision scenarios as important directions for future work (see lines 409-414).

- The robustness of the results to parameter choices, including collision-zone assumptions and the risk-aversion parameter, is not systematically explored. A sensitivity analysis would strengthen confidence in the framework.

We thank the reviewer for this comment and agree that robustness to parameter choices is an important consideration. We note, however, that the manuscript already includes several analysis that address the robustness of the framework, and we have clarified these points in the revised text.

First, in Figure S2, we repeated the simulations shown in Fig. 4 using alternative formulations of the collision-probability function, including exponential and Gaussian cumulative profiles. Despite substantial differences in the shape of these functions, the resulting trajectories were qualitatively similar, indicating that the model's behavior does not depend critically on the specific functional form used to represent collision probability. This robustness arises because

the trajectories are generated through the cumulative integration of relative desirability over time and are therefore influenced primarily by the overall spatial structure of obstacle-related risk rather than by the precise shape of the probability function.

Second, the tolerance boundary serves only to define the obstacle interaction region and is not directly involved in the relative desirability computation itself. Consequently, alternative empirical or obstacle-specific boundary definitions could be incorporated without requiring modification of the underlying decision-making framework.

Third, the risk-aversion parameter α enters the model as a single weighting factor between 0 and 1 that monotonically controls the balance between approach and avoidance. The systematic change in trajectory deviation observed across obstacle sizes in Figure 4 illustrates the behavioral consequences of varying this balance.

More generally, one of the advantages of the present framework is that its qualitative behavior emerges from the interaction between approach and avoidance processes rather than from fine tuning of individual parameter values. The analyses presented in Figure S2 and Figure 4 suggest that the principal behavioral phenomena reported here are robust to reasonable variations in key model assumptions.

Overall, we agree that a comprehensive numerical sensitivity analysis would provide additional insight. However, because the present framework was developed as a systems-level model and was not calibrated to a specific behavioral dataset, the interpretation of parameter sensitivity remains somewhat limited in the absence of an empirical reference against which changes in model behavior can be evaluated. We therefore view a systematic sensitivity analysis as most informative in the context of future studies that fit the model to specific experimental data (please see lines 396-409, 414-418, 429-434).

- The model is presented as generalizable to multiple targets and obstacles, but the demonstrated simulations remain relatively simple. More complex cluttered scenarios would improve the manuscript's impact.

We thank the reviewer for this comment. We agree that demonstrations in more densely cluttered environments would be an interesting extension of the present work. However, we would emphasize that the generality of the framework is established primarily at the level of its formulation rather than through exhaustive simulation. Specifically, the mixture policy is defined for arbitrary numbers of targets and obstacles (Eq. 4), and the ability to select among multiple competing targets was demonstrated in the foundational framework on which the present study builds (Christopoulos & Schrater, 2015).

The primary contribution of the current work is the extension of that framework to obstacle avoidance. Accordingly, we focused our simulations on characterizing the novel obstacle-related computations introduced by the model. These demonstrations include not only static obstacles of varying sizes and positions, but also a dynamic scenario in which the target changes location during movement and a new obstacle appears on the updated path (Figure 6). Thus, the

simulations already illustrate the framework's ability to adapt online to changing environmental conditions rather than merely generating trajectories in static settings.

More importantly, the purpose of the simulations was to isolate and understand the fundamental computational interaction between goal-directed and avoidance-related influences. Introducing many simultaneous targets and obstacles would substantially increase the complexity of the environment, but would also make it more difficult to identify the specific mechanisms responsible for the resulting behavior. We therefore chose simplified scenarios deliberately, as is common when introducing a new computational framework, to establish the core principles before considering more complex environments.

That said, we agree that scenarios involving multiple simultaneous targets and obstacles represent an important next step. Indeed, such environments may provide a particularly informative setting in which to evaluate the model, because the dynamic weighting and competition among multiple policies become increasingly consequential as environmental complexity grows. We have revised the manuscript to make explicit that the model's generality derives from its mathematical formulation and that demonstrations in more complex cluttered environments remain an important direction for future work(see lines 419-429).

Minor points:

- The manuscript could be improved by condensing repeated conceptual explanations and streamlining some sections for clarity and conciseness.

We thank the reviewer for this suggestion. We have condensed repeated conceptual material and streamlined the manuscript accordingly, primarily in the “A computational model for selecting action plans in complex environments” subsection of the Results section and the “Model architecture” and “Computing target and obstacle policy desirability” subsections of the Methods section(see lines 155-156, 471-477, and 588), while keeping the brief restatements needed in the Results section to remain interpretable before the presentation of the Methods section, according to the suggested format of PLoS Computational Biology.

- A few parts of the notation and terminology could be simplified for readability.

We thank the reviewer for this suggestion. We have revised the manuscript to improve consistency and readability. Specifically, we unified the notation for the state and control vectors and the terminal-time symbol across the Results and Methods sections, corrected a typesetting error in the soft-max expression, and assigned distinct symbols to a few quantities that had previously shared one notation (for instance, the velocity vector and the cost-to-go function).

- The manuscript would benefit from a careful language edit.

We thank the reviewer for this suggestion. We have carefully proofread the manuscript and corrected the remaining typographical and grammatical errors throughout the text. We believe these revisions improve the overall readability of the manuscript.

References

1. Cisek P. Cortical mechanisms of action selection: the affordance competition hypothesis. Philosophical Transactions of the Royal Society B: Biological Sciences. 2007 Sep 29;362(1485):1585-99.
2. Christopoulos V, Schrater PR. Dynamic integration of value information into a common probability currency as a theory for flexible decision making. PLoS computational biology. 2015 Sep 22;11(9):e1004402.
3. Christopoulos V, Bonaiuto J, Andersen RA. A biologically plausible computational theory for value integration and action selection in decisions with competing alternatives. PLoS computational biology. 2015 Mar 24;11(3):e1004104.
4. Zhong S, Pouratian N, Christopoulos V. Computational mechanism underlying switching of motor actions. PLoS Computational Biology. 2025 Feb 10;21(2):e1012811.
5. Munoz DP, Everling S. Look away: the anti-saccade task and the voluntary control of eye movement. Nature Reviews Neuroscience. 2004 Mar 1;5(3):218-28.
6. Gail A, Andersen RA. Neural dynamics in monkey parietal reach region reflect context-specific sensorimotor transformations. Journal of Neuroscience. 2006 Sep 13;26(37):9376-84.
7. Gail A, Klaes C, Westendorff S. Implementation of spatial transformation rules for goal-directed reaching via gain modulation in monkey parietal and premotor cortex. Journal of Neuroscience. 2009 Jul 29;29(30):9490-9.
8. Pearce TM, Moran DW. Strategy-dependent encoding of planned arm movements in the dorsal premotor cortex. Science. 2012 Aug 24;337(6097):984-8.
9. Moll L, Kuypers HG. Premotor cortical ablations in monkeys: contralateral changes in visually guided reaching behavior. Science. 1977 Oct 21;198(4314):317-9.
