# Supplementary material for: An integrative computational approach for obstacle avoidance during action selection
Source: PLoS Comput Biol. 2026 Oct 26;22(10):e1013756. doi: 10.1371/journal.pcbi.1013756 (PMC13719039; doi:10.1371/journal.pcbi.1013756)
Supplement: Supplementary file 3 [file pcbi.1013756.s008.pdf]

The manuscript has definitely improved after revisions, and almost all issues pointed out in earlier rounds have been addressed satisfactorily. It is especially nice to see that the authors clarify that the model presented is a systems-level computational approach and not a quantitatively fitted model of a specific behavioral experiment.

We thank the reviewer for the positive assessment of our revised manuscript and for the constructive comments. We have reviewed the manuscript and made further clarifications to the scope of the behavioral comparisons, the computational interpretation of the model, and the limitations of the present simulations. Our responses are provided below, and the revisions are highlighted in blue in the revised manuscript.

My last concern is the relation between replicating qualitatively human behavioral patterns and explaining quantitatively human behavior. The Abstract states that the model "captures key features of human motor behavior". As the simulations have not been quantitatively fitted to behavioral datasets, the authors should ensure that in the Abstract, Author Summary, Results, and Discussion, all claims that refer to human behavior reflect replication of qualitative patterns only.

We thank the reviewer for this comment and agree that the qualitative nature of the comparisons should be reflected consistently throughout the manuscript. Accordingly, we have reviewed the Abstract, Author Summary, Results, and Discussion and revised the relevant claims to distinguish the reproduction of characteristic behavioral patterns from quantitative validation against human data (see page 2, line 28; page 2, line 42; page 2, lines 44-45; page 5, line 111; page 10, lines 233-234; page 12, lines 251-254; page 16, line 348; page 17, lines 376-378; page 17, lines 384-387; page 17, line 389; page 20, lines 457-459).

My comment regarding quantitative validation is justified by the stated scope of the manuscript. Nevertheless, the Discussion section should make it clear that this study does not prove whether this particular model is quantitatively better than the other models of obstacle avoidance. Proposed behavioral measures such as collision rate, maximum lateral deviation, initial deviation angle, minimum clearance, and trajectory curvature can be used as the targets of validation in the future research.

We agree and have clarified in the Limitations and future perspectives subsection that the qualitative comparisons presented here do not demonstrate quantitative superiority over other obstacle-avoidance models. We have retained the discussion of behavioral measures that could serve as benchmarks for future quantitative evaluation (see page 19, line 430-440).

Clarification of the notion of policy negation is very important. The authors should make sure that throughout the manuscript policy negation will be discussed as a computational tool and not a biological phenomenon. The biological interpretation of the proposed model should stay on the level of the hypothesis that motivates subsequent neurophysiological and perturbation experiments.

We thank the reviewer for emphasizing this distinction. We have revised the Model Characteristics and Biological Plausibility subsection to distinguish the conceptual analogy motivating policy negation from evidence of its neural implementation. We also emphasized in the Limitations and future perspectives subsection that neurophysiological recordings and perturbation experiments are necessary for evaluating the biological interpretation of the model (see page 16, lines 355-356; lines 363-365; page 20, lines 448-450).

Explanation of the goods-related term is much clearer now. The authors should make sure that distinction between the general framework and its particular implementation stays. In the case of presented simulations, the obstacle-related value is mostly defined by collision probability while the target reward is approximately constant. Statements regarding integration of rewards, costs, and risks should be made accordingly.

We agree that the general framework should remain distinct from the implementation examined here. We have revised the Discussion section to emphasize that the simulations combine action-related costs with collision probability while keeping target reward constant (see also page 27, lines 609-610). We have also adjusted the interpretation of collisions in the Results section to avoid implying an explicit comparison between avoidance cost and a separately specified collision loss (see page 14, lines 315-317; page 12, lines 251-254).

I appreciate that the authors demonstrate in Figure S2 that qualitatively correct trajectories can be produced independently of a particular function of collision probability. This should not be referred to as robustness with respect to parameters because the comprehensive analysis of the risk-aversion parameter, tolerance boundary, controller parameters, and other parameters has not been performed yet. The authors should change all statements regarding robustness and explicitly state that robustness was only shown for particular assumptions.

We thank the reviewer for this clarification. We have revised the Results and the caption of Fig. S2 to describe the comparison among the tested collision-probability decay functions without implying general robustness to parameter choices. We have also clarified in the Limitations and future perspectives subsection that a comprehensive sensitivity analysis of the risk-aversion parameter, tolerance boundary, controller parameters, and other model parameters has not been conducted (see page 12, lines 255-259; page 42, lines 859-860; page 18, lines 412-416).

Similar to mathematical formulation that allows for arbitrary number of targets and obstacles, the empirical demonstrations are still relatively simple. The authors should distinguish between scalability of the framework from demonstrated behavioral performance in complex cluttered environments. The latter remains to be shown.

We agree and have clarified in the Limitations and future perspectives subsection that the mathematical ability to accommodate multiple targets and obstacles does not establish behavioral performance in complex cluttered environments. Such performance remains to be evaluated, while the simpler simulations presented here were designed to isolate the interaction between target-approach and obstacle-avoidance processes. We have also revised the Abstract to frame applications

to more complex and naturalistic settings as a direction for future investigation (see page 19, lines 423-424; lines 428-429; page 2, lines 30-33).

The authors should conduct the final consistency and language check. Especially, the authors should be careful when moving between computational description and biological realization in the text. The statements such as "the brain computes" or "the brain combines" or any other similar mechanistic phrases should be avoided if the manuscript only demonstrates the computational sufficiency.

We thank the reviewer for this suggestion. We have reviewed the manuscript for consistency, in particular the distinction between computational descriptions and biological interpretations. In the Author Summary and Discussion sections, we have revised the descriptions of action preparation, policy combination, and trajectory generation to refer to processes within the model, while keeping the neural interpretation hypothetical (see page 2, lines 38-40; page 15, lines 332-336; page 17, lines 384-387).

Taking into consideration that these are relatively minor revisions, I believe that the manuscript represents an interesting extension of the relative-desirability framework for obstacle avoidance. The authors became much clearer about what has been shown in simulations and what should be checked in the future. I do not think that there are any substantial methodological changes necessary at this point.

We thank the reviewer again for the constructive feedback. We believe these revisions address the remaining concerns and improve the clarity and consistency of the manuscript.
