# Supplementary material for: An integrative computational approach for obstacle avoidance during action selection
Source: PLoS Comput Biol. 2026 Oct 26;22(10):e1013756. doi: 10.1371/journal.pcbi.1013756 (PMC13719039; doi:10.1371/journal.pcbi.1013756)
Supplement: Supplementary file 4 [file pcbi.1013756.s004.docx]

**Major Comments**

(Line ~205)

The selection of the tolerance boundary (outer diameter of the expanded collision zone) appears fixed and somewhat arbitrary. While the approach is computationally convenient, prior experimental work suggests that safety margins are not constant but instead vary systematically with obstacle geometry and material properties, reflecting differences in perceived collision risk.

Experimental studies quantifying minimum clearance distance during 3D reaching tasks have shown that clearance depends on obstacle shape, size, and fragility, and that perceived risk can be formalized using probabilistic or Bayesian decision-theoretic frameworks, like Tresilian (1998): Attention in action or obstruction of movement? A kinematic analysis of avoidance behavior in prehension; Trommershäuser et al. (2008): Decision making, movement planning and statistical decision theory; Yang et al. (2021a): A collision avoidance algorithm for human motion prediction based on perceived risk of collision – Part 1: Model development; Yang et al. (2021b): A collision avoidance algorithm for human motion prediction based on perceived risk of collision – Part 2: Application; Baus & Yang (2025): Minimum clearance distance during upper-extremity collision avoidance reaching tasks incorporating perceived risk.

Discussing such findings would help contextualize the choice of tolerance boundaries in the current model and clarify whether the fixed buffer represents a simplification or a principled approximation. At minimum, the authors should explicitly justify the choice of the tolerance boundary and discuss how obstacle-dependent or experimentally informed buffer zones could be incorporated in future extensions.

(Line ~208)

The linear decay of collision probability between the inner and outer boundaries is intuitive. Still, it is unclear whether this assumption is supported by empirical or theoretical work, or whether it was selected primarily for simplicity. Prior studies on risk-sensitive motor control and collision avoidance suggest that perceived risk may scale nonlinearly with distance and uncertainty.

The authors are encouraged to clarify whether alternative decay functions were considered and to discuss how sensitive the model behavior is to this assumption. Even a brief justification or acknowledgment of this limitation would strengthen the modeling rationale.

Results and Discussion:

Throughout the Results and Discussion, the manuscript states that the model behavior is “consistent with human studies.” While this appears qualitatively correct, the validation remains entirely qualitative. This is acceptable, but it should be clearly framed as such.

The authors may consider explicitly acknowledging this limitation and briefly discussing whether simple quantitative metrics (e.g., trajectory curvature, clearance distance, or deviation magnitude) could be extracted for future comparison with experimental data.

(Line ~530)

The current collision probability model assumes obstacles can be represented using concentric circular boundaries. While this is reasonable for proof-of-concept simulations, real-world obstacles often have irregular or anisotropic geometries.

Recent experimental work using full 3D obstacle surfaces and segment-level clearance metrics suggests that obstacle shape plays a critical role in avoidance strategies (e.g., Chapman & Goodale, 2008: Missing in action: the effect of obstacle position and size on avoidance while reaching; Baus & Yang, 2025). A brief discussion of how the framework might be generalized to irregular obstacle geometries would improve the model's scope and realism.

**Minor Comments:**

- In Figure 2 and several subsequent figures, axes lack labels and units. Please add axis names and units for clarity.
- Additionally, please explain why axis limits differ across panels, as this may affect visual comparison.
- Although placing Methods after the Discussion follows PLOS formatting conventions, readers may benefit from earlier access to key modeling details, particularly to aid interpretation of simulated trajectories.
- Line ~436: missing comma.
