# Supplementary material for: An integrative computational approach for obstacle avoidance during action selection
Source: PLoS Comput Biol. 2026 Oct 26;22(10):e1013756. doi: 10.1371/journal.pcbi.1013756 (PMC13719039; doi:10.1371/journal.pcbi.1013756)
Supplement: Peer review history

# Peer review: Decision Letter 0

**DOI:** 10.1371/journal.pcbi.1013756.r001

**Authors:** Joseph Ayers, Varun Dutt

15 Feb 2026

An integrative computational approach for obstacle avoidance during action selection

PLOS Computational Biology

Dear Dr. Christopoulos,

Thank you for submitting your manuscript to PLOS Computational Biology. After careful consideration, we feel that it has merit but does not fully meet PLOS Computational Biology's publication criteria as it currently stands. Therefore, we invite you to submit a revised version of the manuscript that addresses the points raised during the review process.

Please submit your revised manuscript by Apr 17 2026 11:59PM. If you will need more time than this to complete your revisions, please reply to this message or contact the journal office at ploscompbiol@plos.org. When you're ready to submit your revision, log on to https://www.editorialmanager.com/pcompbiol/ and select the 'Submissions Needing Revision' folder to locate your manuscript file.

Please include the following items when submitting your revised manuscript:

* A letter that responds to each point raised by the editor and reviewer(s). You should upload this letter as a separate file labeled 'Response to Reviewers'. This file does not need to include responses to formatting updates and technical items listed in the 'Journal Requirements' section below.

* A marked-up copy of your manuscript that highlights changes made to the original version. You should upload this as a separate file labeled 'Revised Manuscript with Track Changes'.

* An unmarked version of your revised paper without tracked changes. You should upload this as a separate file labeled 'Manuscript'.

If you would like to make changes to your financial disclosure, competing interests statement, or data availability statement, please make these updates within the submission form at the time of resubmission. Guidelines for resubmitting your figure files are available below the reviewer comments at the end of this letter

We look forward to receiving your revised manuscript.

Kind regards,

Varun Dutt, Ph.D

Academic Editor

PLOS Computational Biology

Joseph Ayers

Section Editor

PLOS Computational Biology

**Journal Requirements:**

If the reviewer comments include a recommendation to cite specific previously published works, please review and evaluate these publications to determine whether they are relevant and should be cited. There is no requirement to cite these works unless the editor has indicated otherwise.

1) We ask that a manuscript source file is provided at Revision. Please upload your manuscript file as a .doc, .docx, .rtf or .tex. If you are providing a .tex file, please upload it under the item type u2018LaTeX Source Fileu2019 and leave your .pdf version as the item type u2018Manuscriptu2019.

2) Please upload all main figures as separate Figure files in .tif or .eps format. For more information about how to convert and format your figure files please see our guidelines:

https://journals.plos.org/ploscompbiol/s/figures

3) We have noticed that you have uploaded Supporting Information files, but you have not included a list of legends. Please add a full list of legends for your Supporting Information files after the references list.

4) Some material included in your submission may be copyrighted. According to PLOSu2019s copyright policy, authors who use figures or other material (e.g., graphics, clipart, maps) from another author or copyright holder must demonstrate or obtain permission to publish this material under the Creative Commons Attribution 4.0 International (CC BY 4.0) License used by PLOS journals. Please closely review the details of PLOSu2019s copyright requirements here: PLOS Licenses and Copyright. If you need to request permissions from a copyright holder, you may use PLOS's Copyright Content Permission form.

Please respond directly to this email and provide any known details concerning your material's license terms and permissions required for reuse, even if you have not yet obtained copyright permissions or are unsure of your material's copyright compatibility. Once you have responded and addressed all other outstanding technical requirements, you may resubmit your manuscript within Editorial Manager.

Potential Copyright Issues:

i) Figure 1. Please confirm whether you drew the images / clip-art within the figure panels by hand. If you did not draw the images, please provide (a) a link to the source of the images or icons and their license / terms of use; or (b) written permission from the copyright holder to publish the images or icons under our CC BY 4.0 license. Alternatively, you may replace the images with open source alternatives. See these open source resources you may use to replace images / clip-art:

- https://commons.wikimedia.org

- https://openclipart.org/.

5) In the online submission form, you indicated that Data will be made available upon a reasonable request and after signing a formal data sharing agreement.. All PLOS journals now require all data underlying the findings described in their manuscript to be freely available to other researchers, either

1. In a public repository

2. Within the manuscript itself

3. Uploaded as supplementary information.

This policy applies to all data except where public deposition would breach compliance with the protocol approved by your research ethics board. If your data cannot be made publicly available for ethical or legal reasons (e.g., public availability would compromise patient privacy), please explain your reasons by return email and your exemption request will be escalated to the editor for approval. Your exemption request will be handled independently and will not hold up the peer review process, but will need to be resolved should your manuscript be accepted for publication. One of the Editorial team will then be in touch if there are any issues.

6) Please amend your detailed Financial Disclosure statement. This is published with the article. It must therefore be completed in full sentences and contain the exact wording you wish to be published.

1) State the initials, alongside each funding source, of each author to receive each grant. For example: "This work was supported by the National Institutes of Health (####### to AM; ###### to CJ) and the National Science Foundation (###### to AM)."

2) State what role the funders took in the study. If the funders had no role in your study, please state: "The funders had no role in study design, data collection and analysis, decision to publish, or preparation of the manuscript."

3) If any authors received a salary from any of your funders, please state which authors and which funders..

If you did not receive any funding for this study, please simply state: u201cThe authors received no specific funding for this work.u201d

**Reviewers' comments:**

Reviewer's Responses to Questions

**Comments to the Authors:**

**Please note here if the review is uploaded as an attachment.**

Reviewer #1: the review is uploaded as an attachment

Reviewer #2: This manuscript presents a computational model for action selection in cluttered environments, extending the relative desirability framework (Christopoulos & Schrater, 2015) to incorporate obstacle avoidance. The authors demonstrate through simulations that the model captures qualitative features of human reaching behavior, including trajectory deviations around obstacles and the transient tendency to initiate movements toward obstacles before avoidance.

The paper addresses an important problem in motor control and presents a theoretically interesting framework. The extension of relative desirability to obstacle avoidance is novel, and the biological plausibility argument is well-articulated. However, addressing the concerns outlined below would strengthen the manuscript and improve its suitability for publication.

General comments:

1. The paper relies entirely on qualitative comparisons with previously published human studies. The manuscript would be significantly strengthened by quantitative comparisons with human behavioral data from cited studies using statistical metrics comparing model predictions to human performance.

2. The model's ability to produce trajectories that initially deviate toward obstacles (Figure 5) is presented as a key success. It is unclear how the policy mixture produces initial motion toward the obstacle. This is a critical gap because this phenomenon is highlighted as evidence that the model captures human behavior.

3. No algorithm or pseudocode explains how simulations were performed. Readers cannot understand or reproduce the trajectory generation process.

Additional suggestions:

1. The "75% completion" criterion for velocity magnitude (line 174) needs justification.

2. Figures 2-3: Velocity arrows are invisible at normal viewing size. Either increase arrow size or add an inset showing magnified arrows with explanation.

3. Figure 4: Obstacle sizes described as "small," "medium," "large" should be specified numerically

4. How was movement duration T determined for each simulation? Is it fixed or computed dynamically?

5. The "rim" artifact in Figure 2D is acknowledged but unresolved. Does this artifact affect any trajectory predictions, and if not, why not?

6. What noise model was used to generate trial-to-trial variability? What are the noise parameters?

**********

**Have the authors made all data and (if applicable) computational code underlying the findings in their manuscript fully available?**

The PLOS Data policy requires authors to make all data and code underlying the findings described in their manuscript fully available without restriction, with rare exception (please refer to the Data Availability Statement in the manuscript PDF file). The data and code should be provided as part of the manuscript or its supporting information, or deposited to a public repository. For example, in addition to summary statistics, the data points behind means, medians and variance measures should be available. If there are restrictions on publicly sharing data or code —e.g. participant privacy or use of data from a third party—those must be specified.

Reviewer #1: None

Reviewer #2: **No:**  No. The authors state that "data will be made available upon a reasonable request and after signing a formal data sharing agreement"

**********

PLOS authors have the option to publish the peer review history of their article (what does this mean?). If published, this will include your full peer review and any attached files.

If you choose “no”, your identity will remain anonymous but your review may still be made public.

**Do you want your identity to be public for this peer review?**  For information about this choice, including consent withdrawal, please see our Privacy Policy.

Reviewer #1: No

Reviewer #2: No

[NOTE: If reviewer comments were submitted as an attachment file, they will be attached to this email and accessible via the submission site. Please log into your account, locate the manuscript record, and check for the action link "View Attachments". If this link does not appear, there are no attachment files.]

**Figure resubmission:**

After uploading your figures to PLOS’s NAAS tool - https://ngplosjournals.pagemajik.ai/artanalysis, NAAS will process the files provided and display the results in the "Uploaded Files" section of the page as the processing is complete. If the uploaded figures meet our requirements (or NAAS is able to fix the files to meet our requirements), the figure will be marked as "fixed" above. If NAAS is unable to fix the files, a red "failed" label will appear above. When NAAS has confirmed that the figure files meet our requirements, please download the file via the download option, and include these NAAS processed figure files when submitting your revised manuscript.

**Reproducibility:**

To enhance the reproducibility of your results, we recommend that authors of applicable studies deposit laboratory protocols in protocols.io, where a protocol can be assigned its own identifier (DOI) such that it can be cited independently in the future. Additionally, PLOS ONE offers an option to publish peer-reviewed clinical study protocols. Read more information on sharing protocols at https://plos.org/protocols?utm_medium=editorial-email&utm_source=authorletters&utm_campaign=protocols

Attachment Submitted filename: An integrative computational approach Review.docx

# Author comment: Author response to Decision Letter 1

**DOI:** 10.1371/journal.pcbi.1013756.r002

23 Mar 2026

Attachment Submitted filename: Response to reviewers.pdf

# Peer review: Decision Letter 1

**DOI:** 10.1371/journal.pcbi.1013756.r003

**Authors:** Varun Dutt, Tianming Yang

22 Apr 2026

PCOMPBIOL-D-25-02410R1

An integrative computational approach for obstacle avoidance during action selection

PLOS Computational Biology

Dear Dr. Christopoulos,

Thank you for submitting your manuscript to PLOS Computational Biology. After careful consideration, we feel that it has merit but does not fully meet PLOS Computational Biology's publication criteria as it currently stands. Therefore, we invite you to submit a revised version of the manuscript that addresses the points raised during the review process.

Please submit your revised manuscript by Jun 22 2026 11:59PM. If you will need more time than this to complete your revisions, please reply to this message or contact the journal office at ploscompbiol@plos.org. When you're ready to submit your revision, log on to https://www.editorialmanager.com/pcompbiol/ and select the 'Submissions Needing Revision' folder to locate your manuscript file.

Please include the following items when submitting your revised manuscript:

* A letter that responds to each point raised by the editor and reviewer(s). You should upload this letter as a separate file labeled 'Response to Reviewers'. This file does not need to include responses to formatting updates and technical items listed in the 'Journal Requirements' section below.

* A marked-up copy of your manuscript that highlights changes made to the original version. You should upload this as a separate file labeled 'Revised Manuscript with Track Changes'.

* An unmarked version of your revised paper without tracked changes. You should upload this as a separate file labeled 'Manuscript'.

If you would like to make changes to your financial disclosure, competing interests statement, or data availability statement, please make these updates within the submission form at the time of resubmission. Guidelines for resubmitting your figure files are available below the reviewer comments at the end of this letter.

We look forward to receiving your revised manuscript.

Kind regards,

Varun Dutt, Ph.D

Academic Editor

PLOS Computational Biology

Joseph Ayers

Section Editor

PLOS Computational Biology

**Additional Editor Comments:**

Based upon the referee comments, the manuscript needs a minor revision.

**Journal Requirements:**

When completing the data availability statement of the submission form, you indicated that you will make your data available on acceptance. We strongly recommend all authors decide on a data sharing plan before acceptance, as the process can be lengthy and hold up publication timelines. Please note that, though access restrictions are acceptable now, your entire data will need to be made freely accessible if your manuscript is accepted for publication. This policy applies to all data except where public deposition would breach compliance with the protocol approved by your research ethics board. If you are unable to adhere to our open data policy, please kindly revise your statement to explain your reasoning and we will seek the editor's input on an exemption. Please be assured that, once you have provided your new statement, the assessment of your exemption will not hold up the peer review process.

**Reviewers' comments:**

Reviewer's Responses to Questions

**Comments to the Authors:**

**Please note here if the review is uploaded as an attachment.**

Reviewer #3: This a remarkable contribution to the field of computational modeling of action. The paper has been adequately revised according to the comments of previous reviewers, which has clarified substantially the results and highlighted their relevance to potential practical applications in robotics and other fields. The model is very attractive, conceptually simple, elegantly elaborated, succinctly presented, and nicely illustrated. It will form the foundation of new, exciting research in the fields of motor control, human factors, robotics and other applications.

Reviewer #4: The manuscript by Zhong et al. presents a computational framework for obstacle avoidance during action selection that extends a relative-desirability model of multi-target choice to cluttered reaching environments. The authors propose that target-approach and obstacle-avoidance policies are generated in parallel by stochastic optimal controllers and then combined according to a common probabilistic currency, termed relative desirability. Through simulations, the model reproduces several qualitative features reported in human reaching behavior, including trajectory deviations as a function of obstacle position and size, as well as transient initial movements toward an obstacle before later avoidance. The work is conceptually interesting and offers a biologically motivated perspective on how approach and avoidance tendencies may be dynamically integrated during movement planning.

Major points:

- The manuscript is well motivated and conceptually appealing, but the validation is largely qualitative. The model would be substantially strengthened by direct quantitative comparison to human behavioral data and/or formal comparison with alternative computational accounts.

- The central “policy negation” step is clearly acknowledged as an approximation, but its biological plausibility remains somewhat speculative. This aspect would benefit from deeper mechanistic justification or more cautious framing.

- Although the paper is framed as integrating rewards, costs, and risks, the implemented goods-related term is simplified largely to collision probability. The manuscript should clarify this reduced scope more explicitly.

- The robustness of the results to parameter choices, including collision-zone assumptions and the risk-aversion parameter, is not systematically explored. A sensitivity analysis would strengthen confidence in the framework.

- The model is presented as generalizable to multiple targets and obstacles, but the demonstrated simulations remain relatively simple. More complex cluttered scenarios would improve the manuscript’s impact.

Minor points:

- The manuscript could be improved by condensing repeated conceptual explanations and streamlining some sections for clarity and conciseness.

- A few parts of the notation and terminology could be simplified for readability.

- The manuscript would benefit from a careful language edit.

**********

**Have the authors made all data and (if applicable) computational code underlying the findings in their manuscript fully available?**

The PLOS Data policy requires authors to make all data and code underlying the findings described in their manuscript fully available without restriction, with rare exception (please refer to the Data Availability Statement in the manuscript PDF file). The data and code should be provided as part of the manuscript or its supporting information, or deposited to a public repository. For example, in addition to summary statistics, the data points behind means, medians and variance measures should be available. If there are restrictions on publicly sharing data or code —e.g. participant privacy or use of data from a third party—those must be specified.

Reviewer #3: Yes

Reviewer #4: **No:**  Their "data and code availability" section does not mention code at all and data only very restrictively.

**********

PLOS authors have the option to publish the peer review history of their article (what does this mean?). If published, this will include your full peer review and any attached files.

If you choose “no”, your identity will remain anonymous but your review may still be made public.

**Do you want your identity to be public for this peer review?**  For information about this choice, including consent withdrawal, please see our Privacy Policy.

Reviewer #3: No

Reviewer #4: No

[NOTE: If reviewer comments were submitted as an attachment file, they will be attached to this email and accessible via the submission site. Please log into your account, locate the manuscript record, and check for the action link "View Attachments". If this link does not appear, there are no attachment files.]

**Figure resubmission:**

**Reproducibility:**

To enhance the reproducibility of your results, we recommend that authors of applicable studies deposit laboratory protocols in protocols.io, where a protocol can be assigned its own identifier (DOI) such that it can be cited independently in the future. Additionally, PLOS ONE offers an option to publish peer-reviewed clinical study protocols. Read more information on sharing protocols at https://plos.org/protocols?utm_medium=editorial-email&utm_source=authorletters&utm_campaign=protocols

# Author comment: Author response to Decision Letter 2

**DOI:** 10.1371/journal.pcbi.1013756.r004

12 Jun 2026

Attachment Submitted filename: Response_to_Reviewers_auresp_2.pdf

# Editor report: Decision Letter 2

**DOI:** 10.1371/journal.pcbi.1013756.r005

**Authors:** Varun Dutt, Tianming Yang

17 Sep 2026

PCOMPBIOL-D-25-02410R2

An integrative computational approach for obstacle avoidance during action selection

PLOS Computational Biology

Dear Dr. Christopoulos,

Thank you for submitting your manuscript to PLOS Computational Biology. After careful consideration, we feel that it has merit but does not fully meet PLOS Computational Biology's publication criteria as it currently stands. Therefore, we invite you to submit a revised version of the manuscript that addresses the points raised during the review process.

Please submit your revised manuscript by Nov 17 2026 11:59PM. If you will need more time than this to complete your revisions, please reply to this message or contact the journal office at ploscompbiol@plos.org. When you're ready to submit your revision, log on to https://www.editorialmanager.com/pcompbiol/ and select the 'Submissions Needing Revision' folder to locate your manuscript file.

Please include the following items when submitting your revised manuscript:

* A letter that responds to each point raised by the editor and reviewer(s). You should upload this letter as a separate file labeled 'Response to Reviewers'. This file does not need to include responses to formatting updates and technical items listed in the 'Journal Requirements' section below.

* A marked-up copy of your manuscript that highlights changes made to the original version. You should upload this as a separate file labeled 'Revised Manuscript with Track Changes'.

* An unmarked version of your revised paper without tracked changes. You should upload this as a separate file labeled 'Manuscript'.

If you would like to make changes to your financial disclosure, competing interests statement, or data availability statement, please make these updates within the submission form at the time of resubmission. Guidelines for resubmitting your figure files are available below the reviewer comments at the end of this letter.

As the corresponding author, your ORCID iD is verified in the submission system and will appear in the published article. PLOS supports the use of ORCID, and we encourage all coauthors to register for an ORCID iD and use it as well. Please encourage your coauthors to verify their ORCID iD within the submission system before final acceptance, as unverified ORCID iDs will not appear in the published article. Only the individual author can complete the verification step; PLOS staff cannot verify ORCID iDs on behalf of authors.

We look forward to receiving your revised manuscript.

Kind regards,

Varun Dutt, Ph.D

Academic Editor

PLOS Computational Biology

Joseph Ayers

Section Editor

PLOS Computational Biology

**Additional Editor Comments (if provided):**

The manuscript has definitely improved after revisions, and almost all issues pointed out in earlier rounds have been addressed satisfactorily. It is especially nice to see that the authors clarify that the model presented is a systems-level computational approach and not a quantitatively fitted model of a specific behavioral experiment.

My last concern is the relation between replicating qualitatively human behavioral patterns and explaining quantitatively human behavior. The Abstract states that the model "captures key features of human motor behavior". As the simulations have not been quantitatively fitted to behavioral datasets, the authors should ensure that in the Abstract, Author Summary, Results, and Discussion, all claims that refer to human behavior reflect replication of qualitative patterns only.

My comment regarding quantitative validation is justified by the stated scope of the manuscript. Nevertheless, the Discussion section should make it clear that this study does not prove whether this particular model is quantitatively better than the other models of obstacle avoidance. Proposed behavioral measures such as collision rate, maximum lateral deviation, initial deviation angle, minimum clearance, and trajectory curvature can be used as the targets of validation in the future research.

Clarification of the notion of policy negation is very important. The authors should make sure that throughout the manuscript policy negation will be discussed as a computational tool and not a biological phenomenon. The biological interpretation of the proposed model should stay on the level of the hypothesis that motivates subsequent neurophysiological and perturbation experiments.

Explanation of the goods-related term is much clearer now. The authors should make sure that distinction between the general framework and its particular implementation stays. In the case of presented simulations, the obstacle-related value is mostly defined by collision probability while the target reward is approximately constant. Statements regarding integration of rewards, costs, and risks should be made accordingly.

I appreciate that the authors demonstrate in Figure S2 that qualitatively correct trajectories can be produced independently of a particular function of collision probability. This should not be referred to as robustness with respect to parameters because the comprehensive analysis of the risk-aversion parameter, tolerance boundary, controller parameters, and other parameters has not been performed yet. The authors should change all statements regarding robustness and explicitly state that robustness was only shown for particular assumptions.

Similar to mathematical formulation that allows for arbitrary number of targets and obstacles, the empirical demonstrations are still relatively simple. The authors should distinguish between scalability of the framework from demonstrated behavioral performance in complex cluttered environments. The latter remains to be shown.

The authors should conduct the final consistency and language check. Especially, the authors should be careful when moving between computational description and biological realization in the text. The statements such as "the brain computes" or "the brain combines" or any other similar mechanistic phrases should be avoided if the manuscript only demonstrates the computational sufficiency.

Taking into consideration that these are relatively minor revisions, I believe that the manuscript represents an interesting extension of the relative-desirability framework for obstacle avoidance. The authors became much clearer about what has been shown in simulations and what should be checked in the future. I do not think that there are any substantial methodological changes necessary at this point.

**Journal Requirements:**

If the reviewer comments include a recommendation to cite specific previously published works, please review and evaluate these publications to determine whether they are relevant and should be cited. There is no requirement to cite these works unless the editor has indicated otherwise.

1) In the online submission form, you indicated that your data will be submitted to a repository upon acceptance. We strongly recommend all authors deposit their data before acceptance, as the process can be lengthy and hold up publication timelines. Please note that, though access restrictions are acceptable now, your entire minimal dataset will need to be made freely accessible if your manuscript is accepted for publication. This policy applies to all data except where public deposition would breach compliance with the protocol approved by your research ethics board. If you are unable to adhere to our open data policy, please kindly revise your statement to explain your reasoning and we will seek the editor's input on an exemption.

**Reviewers' comments:**

[NOTE: If reviewer comments were submitted as an attachment file, they will be attached to this email and accessible via the submission site. Please log into your account, locate the manuscript record, and check for the action link "View Attachments". If this link does not appear, there are no attachment files.]

**Figure resubmission:**

**Reproducibility:**

To enhance the reproducibility of your results, we recommend that authors of applicable studies deposit laboratory protocols in protocols.io, where a protocol can be assigned its own identifier (DOI) such that it can be cited independently in the future. Additionally, PLOS ONE offers an option to publish peer-reviewed clinical study protocols. Read more information on sharing protocols at https://plos.org/protocols?utm_medium=editorial-email&utm_source=authorletters&utm_campaign=protocols

# Author comment: Author response to Decision Letter 3

**DOI:** 10.1371/journal.pcbi.1013756.r006

22 Sep 2026

Attachment Submitted filename: Response_to_reviewers_auresp_3.pdf

# Editor report: Decision Letter 3

**DOI:** 10.1371/journal.pcbi.1013756.r007

**Authors:** Varun Dutt, Tianming Yang

12 Oct 2026

Dear DR Christopoulos,

We are pleased to inform you that your manuscript 'An integrative computational approach for obstacle avoidance during action selection' has been provisionally accepted for publication in PLOS Computational Biology.

Before your manuscript can be formally accepted you will need to complete some formatting changes, which you will receive in a follow up email. A member of our team will be in touch with a set of requests.

Please note that your manuscript will not be scheduled for publication until you have made the required changes, so a swift response is appreciated.

IMPORTANT: The editorial review process is now complete. PLOS will only permit corrections to spelling, formatting or significant scientific errors from this point onwards. Requests for major changes, or any which affect the scientific understanding of your work, will cause delays to the publication date of your manuscript.

Should you, your institution's press office or the journal office choose to press release your paper, you will automatically be opted out of early publication. We ask that you notify us now if you or your institution is planning to press release the article. All press must be co-ordinated with PLOS.

Thank you again for supporting Open Access publishing; we are looking forward to publishing your work in PLOS Computational Biology.

Best regards,

Tianming Yang, Ph.D.

Section Editor

PLOS Computational Biology

Joseph Ayers

Section Editor

PLOS Computational Biology

***********************************************************

The authors have satisfactorily addressed the remaining issues raised in the previous round of evaluation. In particular, the revised manuscript now consistently distinguishes qualitative reproduction of characteristic human behavioral patterns from quantitative validation against behavioral data. The authors also explicitly acknowledge that the present study does not establish quantitative superiority over alternative models and identify appropriate behavioral measures for future quantitative validation.

The revised manuscript further clarifies that policy negation is a computational abstraction rather than a proposed biological mechanism, and appropriately frames the biological interpretation of the model as a hypothesis requiring future neurophysiological and perturbation studies. The distinction between the general relative-desirability framework and the specific implementation examined here is also substantially clearer: the present simulations combine action-related costs with collision probability while target reward is held constant.

The authors have appropriately restricted their claims concerning robustness to the collision-probability functions actually examined and explicitly acknowledge that a comprehensive parameter-sensitivity analysis has not been performed. Finally, they now clearly distinguish the mathematical ability of the framework to accommodate multiple targets and obstacles from demonstrated behavioral performance in complex cluttered environments.

I find that these revisions satisfactorily address the remaining concerns. The scope and limitations of the work are now presented appropriately, and I do not see a need for further methodological revision.

I therefore recommend acceptance of the manuscript in its present form, subject to standard editorial and production-level corrections.

# Editor report: Acceptance letter

**DOI:** 10.1371/journal.pcbi.1013756.r008

**Authors:** Varun Dutt, Tianming Yang

PCOMPBIOL-D-25-02410R3

An integrative computational approach for obstacle avoidance during action selection

Dear Dr Christopoulos,

I am pleased to inform you that your manuscript has been formally accepted for publication in PLOS Computational Biology. Your manuscript is now with our production department and you will be notified of the publication date in due course.

The corresponding author will soon be receiving a typeset proof for review, to ensure errors have not been introduced during production. Please review the PDF proof of your manuscript carefully, as this is the last chance to correct any errors. Please note that major changes, or those which affect the scientific understanding of the work, will likely cause delays to the publication date of your manuscript.

Soon after your final files are uploaded, unless you have opted out, the early version of your manuscript will be published online. The date of the early version will be your article's publication date. The final article will be published to the same URL, and all versions of the paper will be accessible to readers.

For Research, Software, and Methods articles, you will receive an invoice from PLOS for your publication fee after your manuscript has reached the completed accept phase. If you receive an email requesting payment before acceptance or for any other service, this may be a phishing scheme. Learn how to identify phishing emails and protect your accounts at https://explore.plos.org/phishing.

Thank you again for supporting PLOS Computational Biology and open-access publishing. We are looking forward to publishing your work!

With kind regards,

Anitha Samidurai

PLOS Computational Biology | Carlyle House, Carlyle Road, Cambridge CB4 3DN | United Kingdom ploscompbiol@plos.org | Phone +44 (0) 1223-442824 | ploscompbiol.org | @PLOSCompBiol
